# Identification of a New Rhoptry Neck Complex RON9/RON10 in the Apicomplexa Parasite *Toxoplasma gondii*

**DOI:** 10.1371/journal.pone.0032457

**Published:** 2012-03-12

**Authors:** Mauld H. Lamarque, Julien Papoin, Anne-Laure Finizio, Gaelle Lentini, Alexander W. Pfaff, Ermanno Candolfi, Jean-François Dubremetz, Maryse Lebrun

**Affiliations:** 1 UMR 5235 CNRS, Université de Montpellier 2, 34095 Montpellier, France; 2 Institut de Parasitologie et de Pathologie Tropicale, EA 4438, Université de Strasbourg, Strasbourg, France; University of Georgia, United States of America

## Abstract

Apicomplexan parasites secrete and inject into the host cell the content of specialized secretory organelles called rhoptries, which take part into critical processes such as host cell invasion and modulation of the host cell immune response. The rhoptries are structurally and functionally divided into two compartments. The apical duct contains rhoptry neck (RON) proteins that are conserved in Apicomplexa and are involved in formation of the moving junction (MJ) driving parasite invasion. The posterior bulb contains rhoptry proteins (ROPs) unique to an individual genus and, once injected in the host cell act as effector proteins to co-opt host processes and modulate parasite growth and virulence. We describe here two new RON proteins of *Toxoplasma gondii*, RON9 and RON10, which form a high molecular mass complex. In contrast to the other RONs described to date, this complex was not detected at the MJ during invasion and therefore was not associated to the MJ complex RON2/4/5/8. Disruptions of either *RON9* or *RON10* gene leads to the retention of the partner in the ER followed by subsequent degradation, suggesting that the RON9/RON10 complex formation is required for proper sorting to the rhoptries. Finally, we show that the absence of RON9/RON10 has no significant impact on the morphology of rhoptry, on the invasion and growth in fibroblasts *in vitro* or on virulence *in vivo*. The conservation of RON9 and RON10 in *Coccidia* and *Cryptosporidia* suggests a specific relation with development in intestinal epithelial cells.

## Introduction

*Toxoplasma gondii* is a protozoan parasite belonging to the phylum Apicomplexa that comprises various parasites responsible for many human and animal diseases such as toxoplasmosis, malaria (*Plasmodium* spp.), or cryptosporidiosis (*Cryptosporidium* spp.). Although asymptomatic in healthy humans, toxoplasmosis might lead to severe complications in firstly-infected pregnant women and immuno-compromised patients. As an obligate intracellular parasite, *T. gondii* actively invades host cells by an actin-myosin-dependent mechanism (for a review [1]) that also requires the coordinated exocytosis of proteins located in apical secretory organelles [2], namely the micronemes and rhoptries which are characteristic of the Apicomplexa phylum (for a review [3]). Successful invasion proceeds through several distinct steps including apical attachment, formation of a moving junction (MJ), progression of the parasite through the junction and concomitant establishment of the parasitophorous vacuole (PV) within which the parasite will further reside and replicate.

Micronemal proteins are mostly adhesins secreted during invasion and then expressed onto the parasite surface and allow motility, recognition and attachment to the host cell through interactions with receptors expressed onto the host cell surface [4]. It has been recently shown that in *P. falciparum*, interaction between the micronemal protein EBA175 and glycophorin A expressed onto the erythrocyte surface is sufficient to trigger rhoptry secretion [5], suggesting that in addition to mediating attachment, this ligand/receptor recognition is the first step of an intracellular signaling cascade mediated by the cytosolic tail of microneme proteins [6] and leading to rhoptry discharge. Rhoptries are club-shaped elongated organelles divided into two distinct suborganellar compartments, the bulbous part and the more anterior thin duct (or neck) through which rhoptry proteins are secreted (for a review [7]) but nothing is known about the determinants responsible for maintaining this shape. A proteomic study of the *T. gondii* rhoptry content led to the identification of about 40 rhoptry proteins, some of which restricted to the bulb (ROPs) and others to the neck (RONs) [8]. Concomitant to the first molecular characterization of RON proteins [8] came the demonstration that RON4 was secreted and localized to the MJ during invasion [9], [10]. The MJ is a tight connection between the parasite and host cell plasma membranes that forms at the apical pole and moves progressively to the posterior end of the parasite as it enters (hence the name “moving junction”). As it serves as an anchor to propel the parasite into the PV, MJ formation is necessary for successful invasion. Although known at the structural level for three decades [11], the MJ molecular composition and organization has been unraveled only recently. It is now well established that its formation relies on the coordinated secretion of both micronemes and rhoptries [9]. Indeed, the micronemal protein AMA1 is secreted and expressed onto the parasite surface, while the rhoptry neck proteins RON2/4/5/8 are secreted into the host cell, with RON2 being inserted as an integral trans-membrane protein into the host plasma membrane allowing a direct interaction with AMA1 [12], [13], [14], while RON4, RON5 and RON8 are translocated beneath the host cell plasma membrane [12]. The secretion of ROP proteins follows RONs discharge [15] but unlike RONs, ROPs are targeted to the PV membrane, to the PV lumen or to the host cell nucleus or cytosol where they hijack the host machinery to modulate the immune response and hence, participate in host cell survival and virulence [16]. ROPs belonging to the ROP2 family have been extensively studied and shown to harbor structural conservation of a protein kinase fold [17]. So far, ROP16 and ROP18 solely have been shown to be active secreted kinases that represent key virulence factors [18], [19], [20], [21].

Rhoptries biogenesis is driven by vesicular trafficking from the Golgi apparatus. Rhoptries are first detected as immature organelles, called pre-rhoptries, which are large vesicles containing a heterogenous dense material, located between the Golgi and the apical area of developing tachyzoites. Several ROPs undergo proteolytic maturation late in the secretory pathway, in the transition step between immature and mature rhoptry. This processing is not a prerequisite for correct targeting of rhoptry proteins [22], [23] or assembly of the RON2/4/5/8 complex [12].

As many rhoptry proteins have been shown to be key players in *T. gondii* invasion, replication and virulence, a better understanding of the rhoptries components along with a characterization of their biological function seems to be crucial. In this study, we have identified two novel rhoptry neck proteins named RON9 and RON10 that are conserved in *Coccidia* and *Cryptosporidia* and form a highly stable hetero-complex distinct from the MJ complex AMA1/RON2/4/5/8. Genetic disruption of *RON9* or *RON10* allowed us to demonstrate that the complex formation is required for proper targeting to the rhoptry neck; a *Δron9*parasite is therefore likely a functional RON9-RON10KO. Furthermore, analysis of the RON9-RON10KO parasites did not detect any defect in development in HFF *in vitro*, and in *T. gondii* RH strain virulence *in vivo*.

## Results

### Monoclonal antibody 2A7 (mAb2A7) recognizes a rhoptry neck antigen

**Figure 1:**
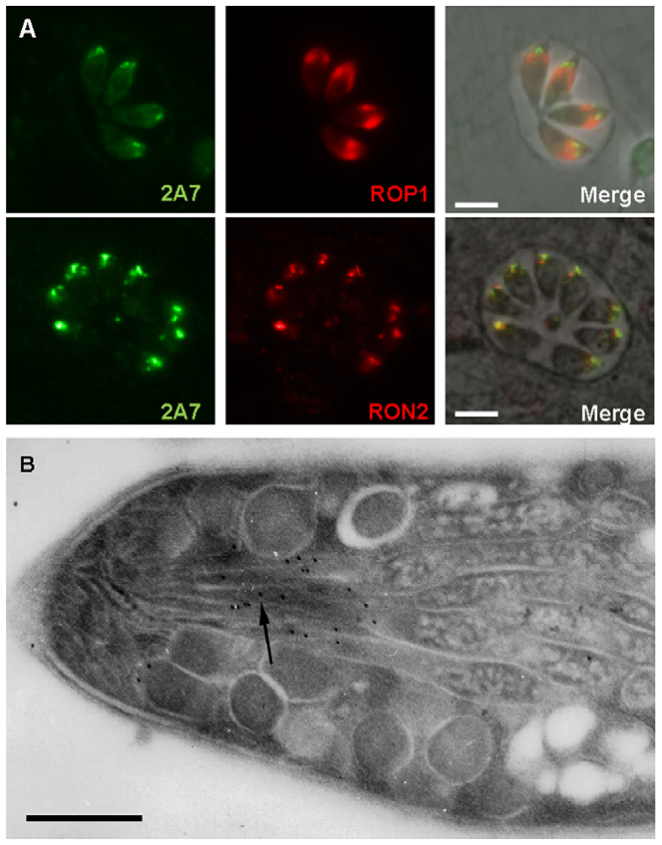
mAb 2A7 recognizes a rhoptry neck protein. (A) Co-immunofluorescence-staining on intracellular tachyzoites with the rhoptry bulb marker anti-ROP1, the rhoptry neck marker RON2 and mAb 2A7. Scale bar = 5 µm. (B) Electron microscopy of *T. gondii* parasites showing the rhoptry neck labeling with mAb 2A7 (black arrow). Scale bar = 0.5 µm.

We investigated the localization of the protein recognized by mAb 2A7 raised against a rhoptry enriched fraction [24]. Immunofluorescence assays (IFAs) carried out on intracellular *T. gondii* tachyzoites showed labeling of the apical pole anterior to the known rhoptry bulb protein ROP1, and colocalization with RON2 staining, suggesting a rhoptry neck localization(Fig. 1A). Immunoelectron microscopy performed on intracellular parasites with mAb 2A7 confirmed this cellular location (Fig. 1B).

### mAb 2A7 reacts with a new rhoptry neck protein named RON9

**Figure 2:**
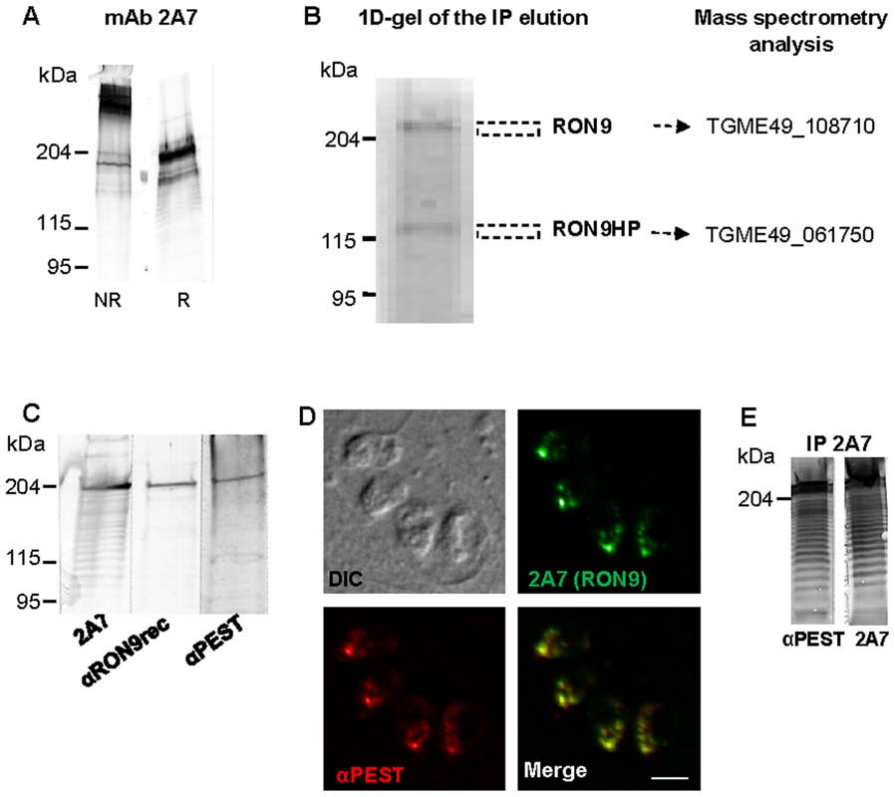
Molecular characterization of RON9. (A) Whole cell lysates of *Δhxgprt* were separated by SDS-PAGE, in non reduced (NR) or reduced (R) conditions, and mAb 2A7 was used to probe the membrane. (B) RON9 and RON9HP were affinity purified with mAb 2A7 from *Δhxgprt* whole cell lysate. Proteins identification was performed by mass spectrometry and subsequent searches against *Toxoplasma* database. (C) Western-blot using mAb 2A7, or anti-RON9rec antibodies produced against a TgRON9 recombinant-GST protein or anti-PEST antibodies produced against the TgRON9 PEST-repetition peptide. The three sera recognize the same protein migrating at a high molecular mass. Scale bar = 5 µm. (D) Co-immunostaining of intracellular *Δhxgprt* parasites with mAb 2A7 and anti-PEST antibodies. (E) Proteins from *Δhxgprt* lysates were immuno-purified using mAb 2A7, then separated by SDS-PAGE and probed with either mAb 2A7 or anti-PEST antibodies.

Immuno-detection with mAb 2A7 on a *T. gondii* tachyzoite lysate in non reduced conditions showed a major band migrating far above the 204 kDa MW marker while in reduced conditions the molecular mass of the protein was found close to 204 kDa (Fig. 2A), suggesting that mAb 2A7 recognized a protein from a homo- or hetero- protein complex linked by disulfide bonds. Based on its cellular location, we named this protein RON9. A set of lower bands showing decreasing intensity and regular spacing was systematically observed, even when freshly egressed parasites were used for SDS-PAGE, which could suggest that RON9 was subjected to proteolysis. The molecular identification of RON9 was confirmed by immuno-affinity chromatography using mAb 2A7 coupled to sepharose-CnBr activated beads and a *T. gondii* soluble lysate. Following extensive washes, proteins were eluted and separated onto a one dimensional acrylamide gel. Silver staining revealed two distinct bands (Fig. 2B), one with a molecular mass corresponding to RON9 and a second one around 140 kDa. Both proteins were in-gel digested with trypsin and the resulting peptides were analyzed by liquid-chromatography coupled to mass spectrometry in an ionic trap. Two peptides corresponding to RON9 were identified, GADVMSQDIR and APIHLAAAPSSFDVVPAK, that matched in the ToxoDB database [25] with the *T. gondii* prediction TGME49_108710 encoding a hypothetical protein of 1108 amino acids with a predicted molecular mass comprised between 122 and 127 kDa. The single peptide recovered from the 140 kDa protein with the sequence SEAEEAIASK matched with the *T. gondii* TGME49_061750 prediction corresponding to a hypothetical protein with a predicted molecular mass of 92 kDa. This protein will be referred hereafter as RON9HP (RON9 hypothetical partner, see above).

Sequencing revealed that RON9 cDNA extends in its 5′ region into the TGME49_108600 prediction located upstream TGME49_108710, and thus leads to the biosynthesis of a 1277 amino acids protein with a predicted molecular weight of 135 kDa and a pI of 4.4 (Figure S1). To confirm that the TGME49_108600 gene encoded the rhoptry neck protein RON9, specific sera were produced. Antibodies were raised either against a GST-recombinant protein comprising the peptides sequences identified in mass spectrometry (anti-RON9rec, see Fig. S1), or against the repeated sequence QANASQSSETPAEENAEEPKQAEE of RON9 (referred here as anti-PEST) described as a PEST repetition by PestFind (http://emboss.bioinformatics.nl/cgi-bin/emboss/epestfind) because of its richness in proline, glutamic acid, serine and threonine residues (see later). In western blot, both the anti-RON9rec and anti-PEST antibodies recognized a protein migrating around 204 kDa in a *T. gondii* lysate (Fig. 2C). By IFA, the anti-PEST serum recognized an antigen co-localizing with mAb 2A7 in intracellular parasites (Fig. 2D), while anti-RON9rec did not react (data not shown). To further confirm the RON9 molecular identification, we performed immuno-purifications using mAb 2A7 and showed that the anti-PEST serum recognized the same protein band as mAb 2A7 (Fig. 2E). Taken together, these results clearly identify RON9 as a 1277 amino acids protein and its coding sequence has been deposited in Genebank under accession number JQ655737.

### Identification of RON10 as a rhoptry neck partner for RON9

**Figure 3:**
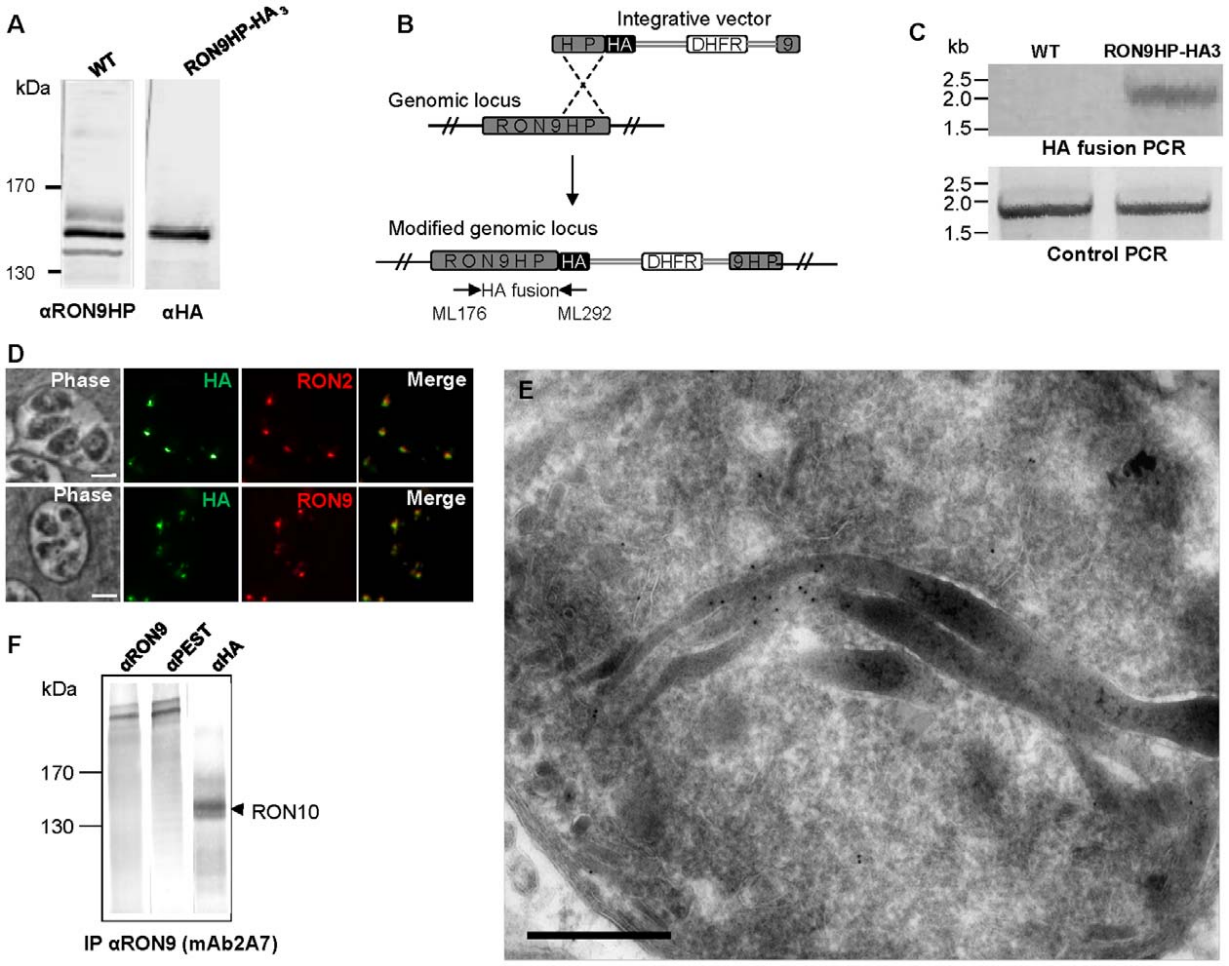
RON9HP is a rhoptry neck protein, renamed RON10. (A) Western-blot carried on *Δku80* cell lysate reveals a major band at 140 kDa with anti-RON9HP antibodies. A protein of the same size is recognized with anti-HA antibodies on RON9-HP-HA_3_ parasites. (B) Scheme depicting the C-terminal HA tagging of the endogenous copy of *RON9HP* (TGME49_061750). A C-terminal fragment of *RON9HP* gene was cloned in frame with an HA epitope in pHA_3_-LIC-DHFR vector. Vector linearization followed by *Δku80* parasites transfection allowed the obtention of a RON9HP-HA_3_ population by single homologous recombination event. (C) To verify the correct genomic integration of the vector into the *RON9HP* locus, PCR reactions were performed on gDNA from the parental *Δku80* strain or RON9HP-HA_3_ parasites using primers ML176 and ML292 depicted by the arrows in (B). PCR allowed amplification of a 2.5 kb product from the recombinant parasites while no amplification was obtained with the parental strain. As a control, amplification of *ATG3* gene [58] was obtained with both gDNA using primers ML339 and ML340. (D) IFAs were carried on RON9HP-HA_3_ parasites with anti-HA antibodies and mAb 2A7 or anti-RON2-4 antibodies. Scale bar = 5 µm. (E) Electron microscopy performed on RON9HP-HA_3_ parasites labelled with anti-HA antibodies shows staining of the rhoptry neck. Scale bar = 0.5 µm. (F) Proteins from RON10-HA_3_ parasites were immuno-purified with mAb 2A7 an run on SDS-PAGE prior to detection using mAb 2A7, anti-PEST or anti-HA antibodies.

We then investigated the molecular identity of the RON9HP protein recovered with RON9 by immuno-affinity (Fig. 2B). Antibodies were raised against a GST-recombinant protein comprising the peptide sequence identified in mass spectrometry (anti-RON9HP) (Fig. S2). In western blot, the anti-RON9HP detected a major band of the expected size (140 kDa) and additional fainter bands (Fig. 3A) and gave in IFA, in addition to a diffuse signal in the cytosol, punctate dots at the apical end of the parasite suggesting a possible rhoptry neck localization (Fig. S3). To bypass the issues of antibodies specificity, we genetically engineered a *T. gondii* strain expressing a HA-tagged version of RON9HP at the endogenous locus, as depicted in figure 3B. The presence of the HA_3_ tag at the C-terminus of *RON9HP* gene (TGME49_061750) was verified by PCR using a forward primer located upstream the C-terminal fragment cloned in the pHA_3_-LIC-DHFR and a reverse primer encompassing the HA_3_ tag. An expected fragment of 2.1 kb was amplified from the gDNA of RON9HP-HA_3_ parasites while no amplification was obtained using the *Δku80* gDNA as a control (Fig. 3C). In western blot, the HA-tagged protein migrated at the same size as the major band revealed by anti-RON9HP (Fig. 3A). To investigate the localization of the RON9HP-HA_3_ protein, IFAs were performed on intracellular tachyzoites with anti-HA antibodies and revealed an apical labeling that perfectly co-localized with the rhoptry neck marker RON2 and also with RON9 (Fig. 3D). This localization was further confirmed by immunoelectron microscopy as shown in figure 3E, allowing us to rename this protein RON10.

**Figure 4:**
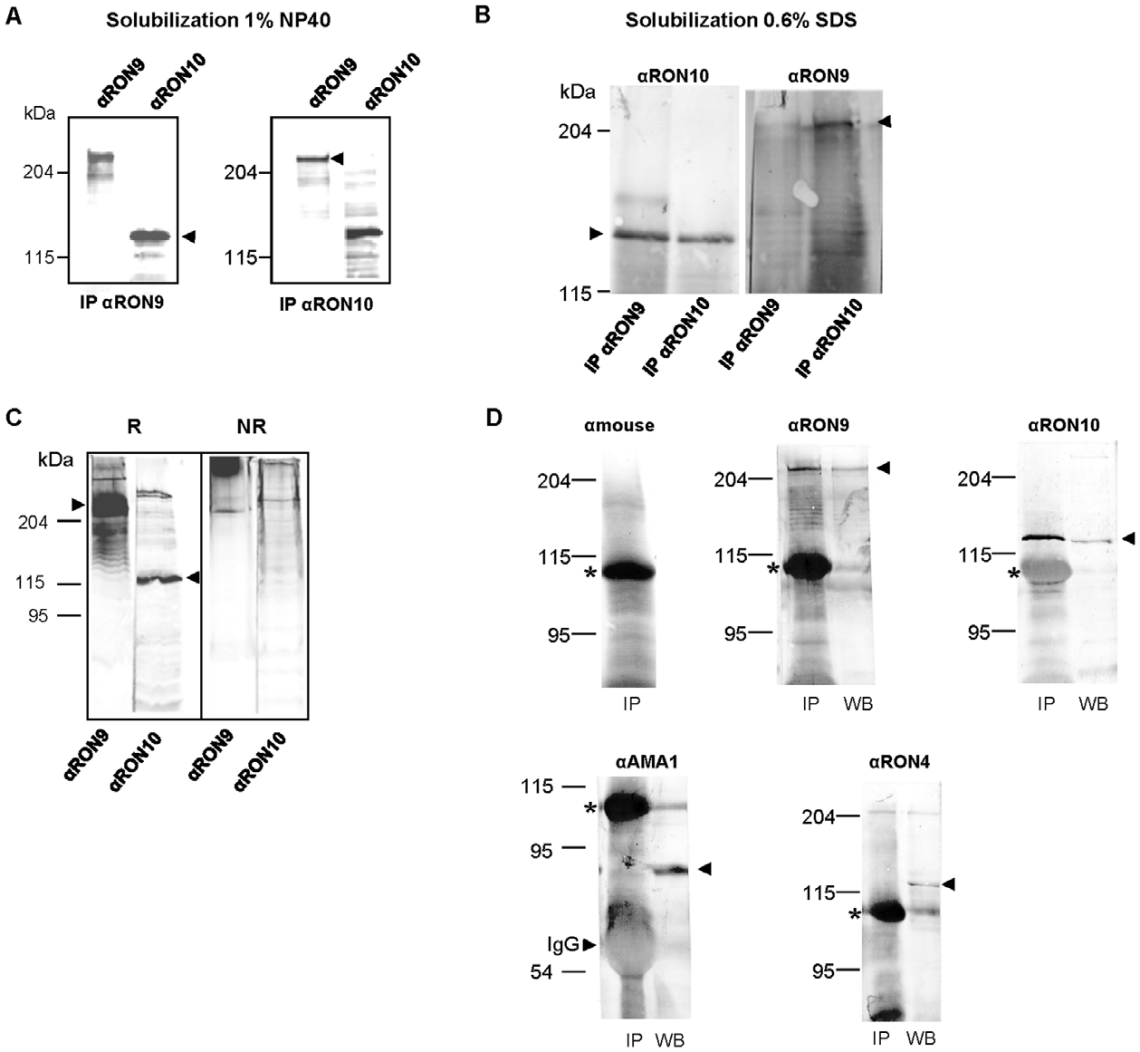
RON9 and RON10 form a highly stable complex, distinct from the AMA1/RON2/4/5/8 junctional complex. Immunopurification assays (A, B and D) were performed on lysates of *Δhxgprt* parasites in the presence of 1% NP40 (A) or 0.6% SDS (B) as described previously [12] with anti-RON9 (IP αRON9) or anti-RON10 (IP αRON10) antibodies. Immuno-purified proteins were detected using the same antibodies, as mentioned on the top of the figure, following SDS-PAGE separation in reduced condition. (C) Whole cell lysates of *Δhxgprt* were separated by SDS-PAGE, in non reduced (NR) or reduced (R) conditions, and mAb 2A7 (αRON9) and αRON10 (αRON9HP) were used to probe the membrane. The arrows indicate the size of the expected protein for each antibody and its corresponding molecular mass. (D) To verify the identification of the proteins co-immunopurified with mAb 2A7 (IP lanes), Western-blot was simultaneously carried out on a tachyzoites lysate (WB lanes) and revealed with secondary anti-mouse alone, mAb 2A7, anti-RON10, anti-AMA1 or anti-RON4 as mentioned on the top. The major band at 110 kDa indicated with an asterisk corresponds to a protein present in the ascitic fluid that binds to protein G-sepharose and was consistently released during the elution step (except when antibodies were CNBr cross-linked to sepharose, as in Fig. 2B).

To further confirm that RON10-HA_3_ was the protein identified in complex with RON9, immuno-purifications were performed on RON10-HA_3_ lysate using mAb 2A7 and revealed in western-blot with the same antibody, or anti-PEST, or anti-HA (Fig. 3F). As expected, RON10-HA_3_ was co-immunopurified along with RON9 protein. Reverse immuno-affinity purifications were also carried out using anti-RON10 antibodies followed by immuno-detection with the anti-RON10 or anti-RON9 antibodies (Fig. 4A). The results indicated that anti-RON10 antibodies were able to co-immunopurify the RON9 protein and that RON10 was recovered after RON9 immuno-purification, thus confirming the RON9-RON10 interaction.

### RON9 and RON10 form a high molecular mass complex, distinct from the RON2/4/5/8-AMA1 junctional complex

Next, we sought to assess the nature of the RON9-RON10 interaction by attempting to disrupt the complex with the use of 0.6% SDS as described previously [12]. As shown in figure 4B, both RON9 and RON10 were still co-immunoprecipitated with anti-RON9 or anti-RON10 antibodies, thereby showing that the binding between these two proteins was highly stable. In addition, immuno-detection revealed that these two proteins did not enter the gel in non reduced conditions while the two proteins were perfectly detected in reduced conditions (Fig. 4C). Taken together, these data suggest that RON9 and RON10 form a stable high molecular complex likely linked *via* disulphide bridges.

We had demonstrated in a previous study the existence at the MJ of a molecular complex formed by the rhoptry neck proteins RON2/4/5/8 and the micronemal protein AMA1 [12]. To determine if the RON9-RON10 complex was part of the junctional RONs-AMA1 complex, *T. gondii* proteins were solubilised under conditions that preserved the MJ complex [10] and proteins were immuno-purified using anti-RON9 antibodies (mAb 2A7) and subsequently revealed with anti-RON9, anti-RON10, anti-AMA1 or anti-RON4 antibodies. Immuno-detection with the same antibodies was performed on tachyzoites lysate to ascertain proteins identification. While anti-RON9 immuno-purified both RON9 and RON10 as expected, AMA1 or RON4 proteins were not detected, suggesting that the RON9-RON10 complex was distinct from the RONs-AMA1 complex. Although not being part of the junctional complex, we examined if RON9 and RON10 would also follow the MJ. For this, we performed IFAs on invading parasites under permeabilization conditions optimized to detect only the material secreted by the parasite [26], but we were unable to detect RON9 and RON10 using the different sera available or anti-HA antibodies on invading RON10-HA_3_ expressing parasites (data not shown). To test whether RON9 and RON10 could be secreted into the host cell, we performed invasion assays in the presence of cytochalasin D, an actin inhibitor, that prevents the parasites from invading the host but still allows rhoptry secretion [27]. These abortive invasions lead to the formation of e-vacuoles that contain secreted material as described previously. While the positive control ROP1 was clearly detected in the e-vacuoles, no RON9 or RON10 staining was observed in the host cell (data not shown).

### Bioinformatic analysis

The *Tg*RON9 sequence harbours an N-terminal predicted signal peptide, as expected for a secreted rhoptry protein, and a predicted C-terminal transmembrane domain (Fig. S1). BLAST analysis showed that RON9 displays orthologues in *Cryptosporidium* (cgd4_2420), *Neospora caninum* (NCLIV_053290) and *Eimeria* (ETH_00015380) only (Fig. S4). While the N-terminal part of the proteins was highly divergent between species, 6 predicted ankyrin domains and one Sushi domain or CCP (complement control protein) were conserved in the C-terminal part of all the RON9 sequences, except CpRON9 that harbours only 5 ankyrin domains (Fig. S1, S4). The ankyrin repeats are a 33 amino-acids motif found in many eukaryotic and prokaryotic proteins and are well described protein-protein interaction domains (for a review [28]). The Sushi domain is found in many complement proteins as well as in adhesion proteins and is characterized by four invariant cysteine residues, an almost invariant tryptophan, as well as highly conserved proline and glycine residues [29]. In *Tg*RON9, all these residues seem to be conserved, except tryptophan. Several internal repeats of 21 amino acids located in the N-terminal part of *Tg*RON9 were detected (Fig. S5) and predicted as PEST sequences (or motifs) using PestFind (http://emboss.bioinformatics.nl/cgi-bin/emboss/epestfind), due to their richness in proline (P), glutamic acid (E), serine (S) and threonine (T) residues. PESTs have been described as targeting signals for protein degradation by the ubiquitin-proteasome system and are found in short-lived proteins [30]. Interestingly, the RON9 orthologues do not display these repeats, except *Nc*RON9 but the repeated sequence is much longer (34 amino acids) and different from that of *Tg*RON9 (Fig. S5).

BLAST analysis showed that *Tg*RON10 displays orthologues in *Cryptosporidium* (cgd8_2530) and *Neospora* (NCLIV_025730) (Fig. S6). *Tg*RON10 also displays a predicted signal peptide in its N-terminus but no known domains or motifs have been identified. The *Cryptosporidium* orthologue contains, as RON9, a Sushi domain and six 29 bp repeats (Fig. S5).

### Disruption of RON9 results in mislocalization of RON10

**Figure 5:**
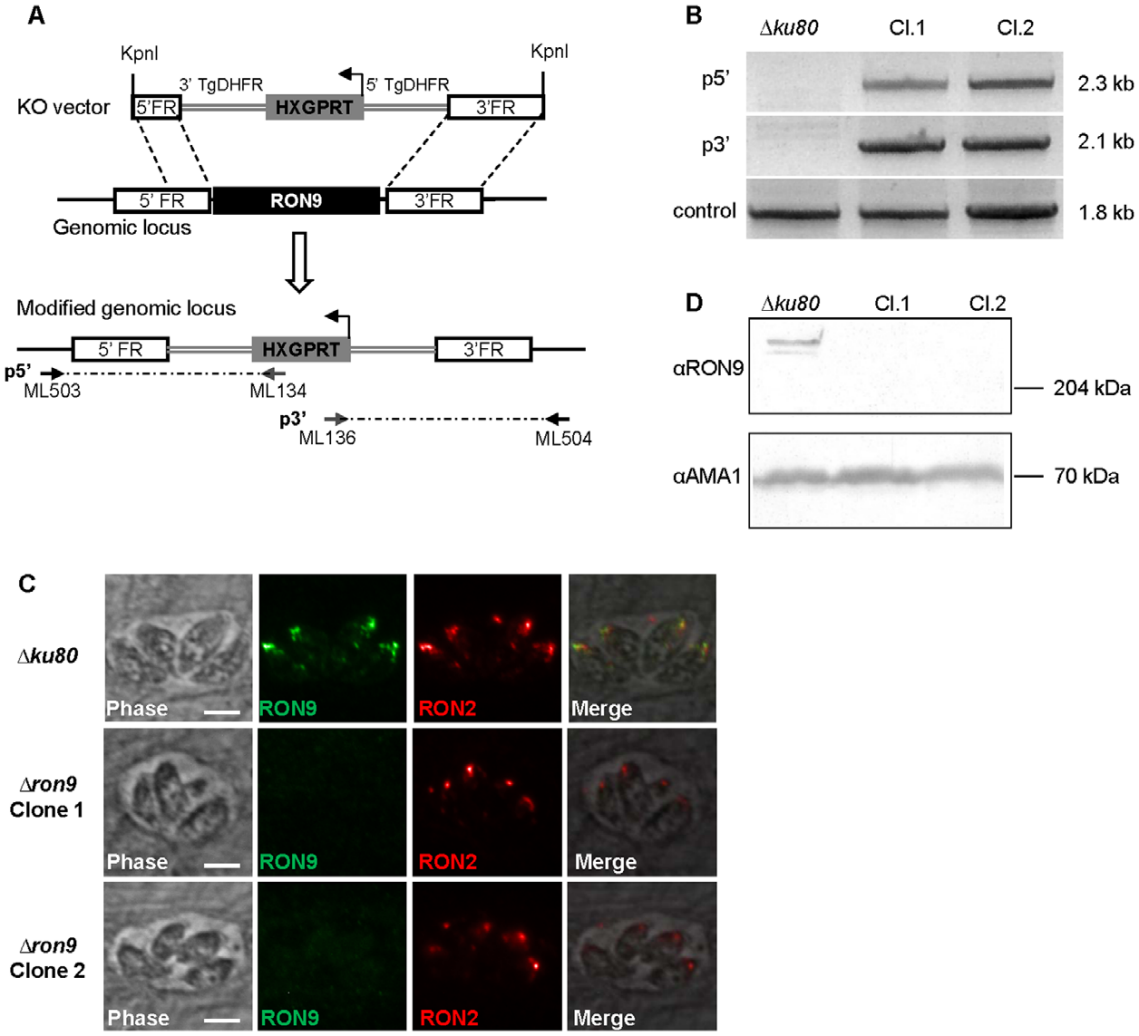
Generation of *Δron9* parasites. (A) Scheme depicting the strategy used to obtain a *Δron9* strain. The 5′ and 3′ flanking regions (FR) of *RON9* were cloned on both sides of *HXGPRT* selection marker, and the vector was linearized with KpnI prior transfection of *Δku80* parasites. Following a double homologous recombination event, *RON9* was replaced by *HXGPRT*. The arrows represent the primers ML503/ML134 and ML136/ML504 used to verify the integration at the 5′ (p5′) and 3′ (p3′) side respectively. (B) PCR reactions to check the vector integration at the RON9 locus were performed on the gDNA of two independent clones of *Δron9* parasites. gDNA of *Δku80* strain was used as a control and amplification of the *ATG3* gene was used as a control of gDNA integrity. As expected, DNA fragments were amplified in the *Δron9* clones with the integration PCR while no DNA could be amplified from the parental strain. Primers ML339 and ML340 in *T. gondii ATG3* gene allowed DNA amplification for the 3 gDNAs tested. (C) mAb 2A7 and anti-RON2 antibodies were used in IFA experiments to verify the absence of expression of RON9 in *Δron9* parasites, compared to the control *Δku80*. Scale bar = 5 µm. (D) Western-blot using mAb 2A7 was performed on control *Δku80* and on *Δron9* parasites, thus confirming the absence of RON9 in the mutant strain. Detection of AMA1 protein was used as a loading control.

We then investigated the role of RON9-RON10 by a genetic disruption approach. To generate a RON9 knock-out parasite line (*Δron9*) in the *Δku80* background, 5′ and 3′ flanking regions of the *RON9* gene were independently cloned on both side of *HXGPRT* resistance marker in the pminiHXGPRT plasmid (Fig. 5A). The resulting construct was transfected in *Δku80* parasites, recombinant parasites were selected, and clonal populations were screened by IFA for the absence of the RON9 protein. Replacement of the *RON9* gene by the *HXGPRT* marker was verified by PCR in the 5′ and 3′ regions using one primer located outside the flanking region and another one in the *HXGPRT* gene, as depicted in figure 5B. As shown in figure 5C in comparison to the *Δku80* strain, RON9 was not detected in the two *Δron9* clones while RON2 was still expressed in the control and *Δron9*populations. Western blot confirmed the absence of RON9 in the *Δron9* parasites as compared to the parental strain (Fig. 5D). Altogether, these results demonstrate the successful disruption of the *RON9* locus and show that RON9 is not essential for *in vitro* propagation of *T. gondii*. As both *Δron9* clones displayed a similar profile, all subsequent analyses were performed with clone 1.

**Figure 6:**
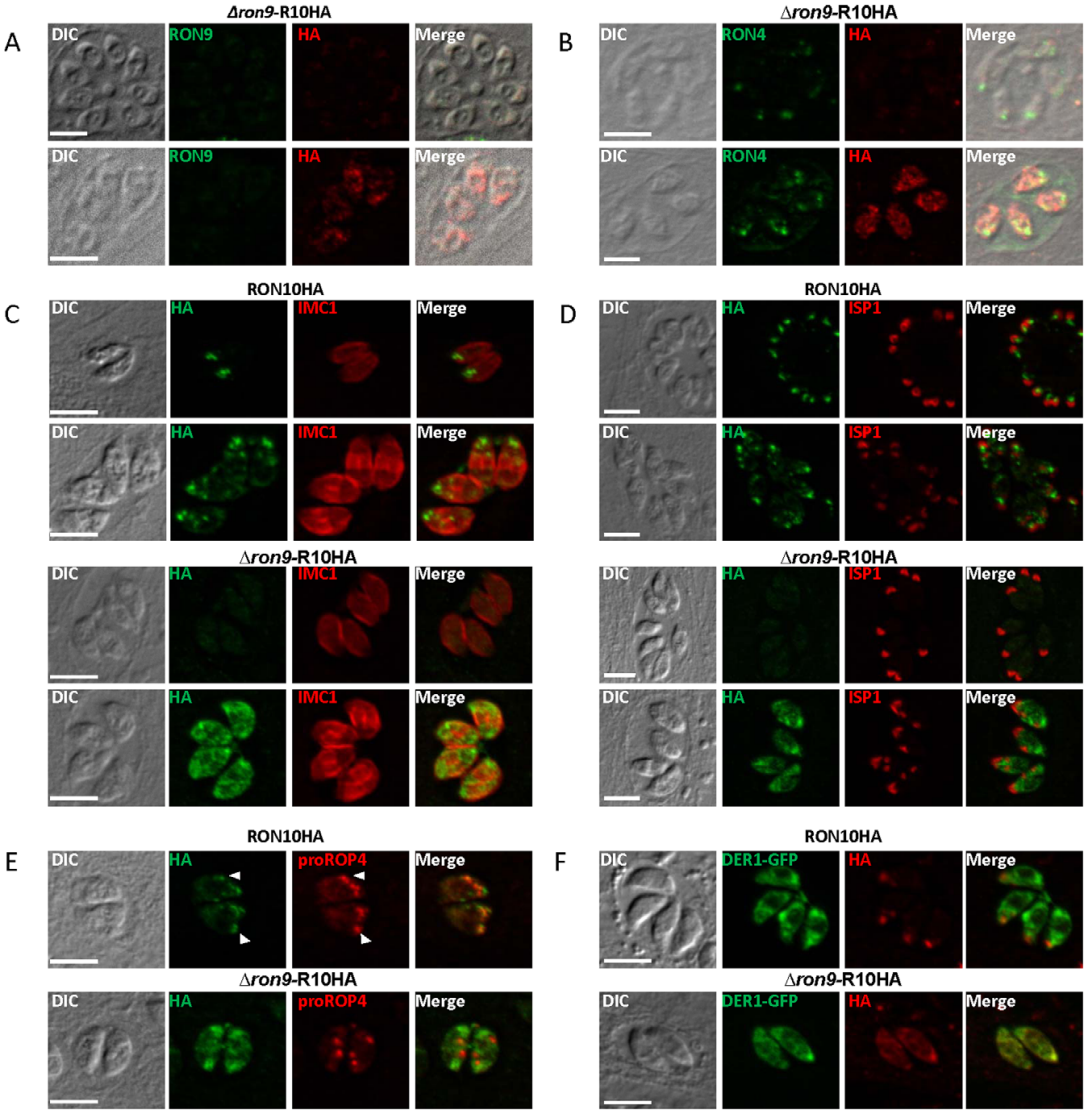
RON10 is mis-targeted in *Δron9* parasites. To follow RON10HA biosynthesis in time and space, co-localization experiments have been performed on *Δron9*-R10HA parasites or RON10HA parasites as a control. Anti-HA antibody was used to follow RON10 fate, while anti-RON9 (A) and anti-RON4 (B) were used to label the rhoptry neck, anti-IMC1 antibodies (C) and anti-ISP1 antibodies (D) allowed us to follow the endodyogeny process during cells replication. Anti-proROP4 antibodies allowed detection of pre-rhoptries (E) and co-transfection of DER1-GFP plasmid allowed detection of the ER (F). Scale bars = 5 µm.

Since we had demonstrated that RON9 and RON10 form a complex, we investigated the cellular location of RON10 in the absence of its RON9 partner. For this purpose, a *Δron9* parasite has been re-engineered in a RON10-HA_3_ background and named *Δron9*-R10HA (Fig. 6A and Fig. S7). IFA performed on *Δron9*-R10HA parasites revealed that RON10 was not associated with the neck of the rhoptry, as demonstrated by the absence of co-localization with RON4 (Fig. 6 B). In most parasites, no staining was observed with an anti-HA antibody (Fig. 6A, top), but in some cases, an intense punctate labeling throughout the cytoplasm but mostly perinuclear was detected (Fig. 6A, bottom). In order to test for a possible stage specificity of the staining, we performed co-immunolabeling with an anti-IMC1 serum, which allows the visualization of the inner membrane complex of both mother and daughter cells during endodyogeny [31]. The punctate RON10 labeling was only observed in parasites undergoing endodyogeny in *Δron9*-R10HA strain, but not in non-dividing parasites (Fig. 6C). A dual labeling with anti-ISP1 antibodies that stain the apical cap of the inner membrane complex in mother and daughter cells [32] confirmed this restricted expression pattern of RON10 to parasites in division (Fig. 6D). Next, to more precisely determine at which step and in which compartment of the secretion pathway RON10 was either retained or mislocalized, we used the anti-proROP4 antibodies [33] as a pre-rhoptry marker that signed the rhoptry biogenesis and transfected the *Δron9*-R10HA strain with the Der1-GFP plasmid [34] to label the ER. While, with parental RON10HA parasites, RON10 was observed in the rhoptry neck of mother cells as well as in discrete new foci corresponding to pre-rhoptries of daughter cells (Fig. 6E), in the *Δron9*-R10HA strain, no co-localization of proROP4 and RON10HA was ever found although both markers were systematically simultaneously observed. In contrast a perfect co-localization with the ER marker was observed (Fig. 6F). All these results showed that RON10 is synthesized at the same time as other rhoptry proteins and correctly routed to the ER, but that in the absence of its partner RON9, RON10 does not reach the pre-rhoptry compartment, stacks in the ER and is probably degraded.

### Disruption of RON10 prevents RON9 trafficking to the rhoptries

**Figure 7:**
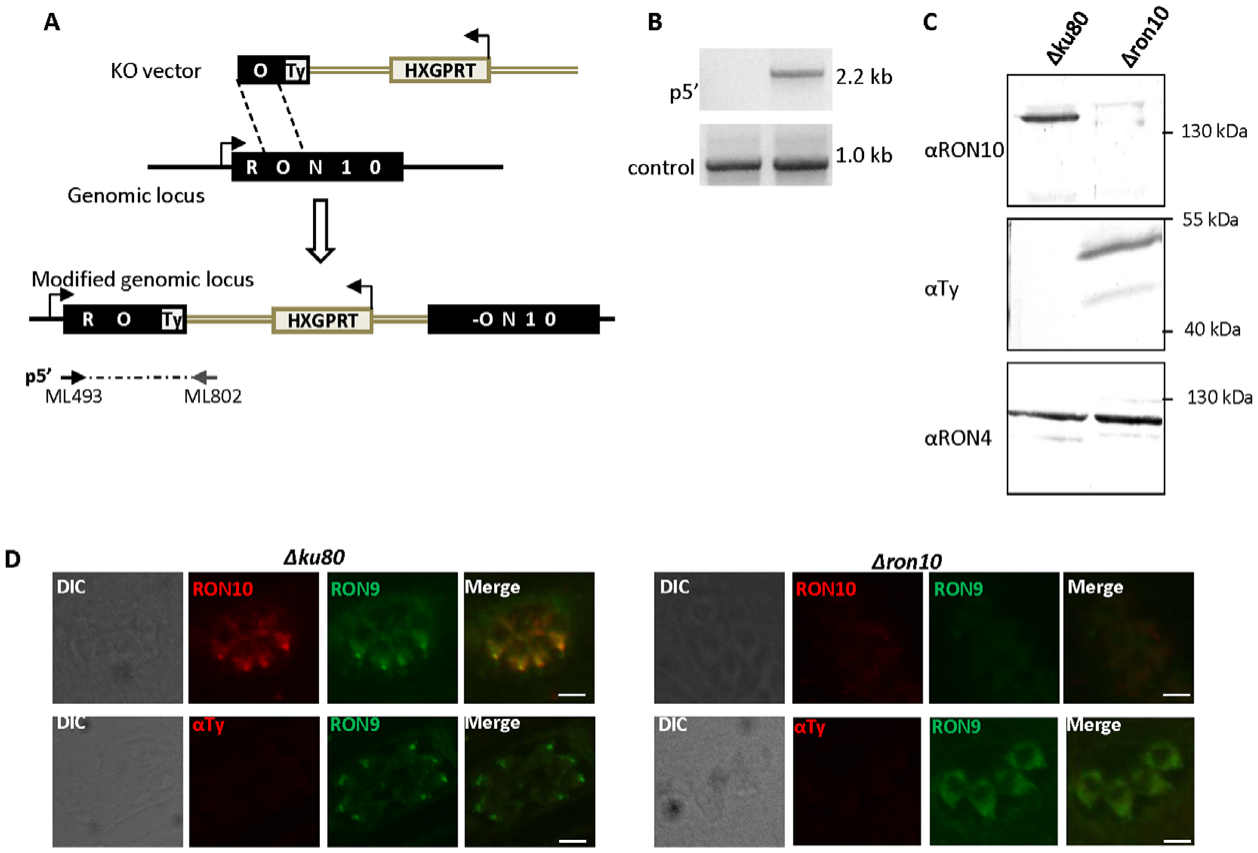
RON9 is mis-localized in *Δron10* parasites. (A) Scheme depicting the *RON10* disruption strategy. Folllowing a single cross-over event, the *RON10* wild type locus is replaced by a truncated version in frame with a Ty tag. The arrows represent the primers ML493/ML802 used to verify the 5′ integration at the *RON10* locus (p5′). (B) The correct vector integration was verified by PCR using primers ML493/ML802 (p5′), and primers ML936/ML937 located in the *RON2* gene were used as a positive control. (C) Western-blot using anti-RON10 antibodies was performed on *Δku80* or *Δron10* parasites, thus confirming the absence of RON10 in the *Δron10* strain. Anti-Ty antibodies show truncated forms of RON10 in the *Δron10* parasites compared to the control, and detection of RON4 was used as a loading control. (D) IFAs were performed on *Δku80* or *Δron10* parasites to follow RON10 and RON9 localizations. Scale bar = 5 µm.

Similarly, we investigated the localization of RON9 in the absence of RON10. We engineered a *Δron10* strain by single homologous cross-over in which the full length *RON10* gene was disrupted and replaced by a truncated version comprising the first 3 exons in frame with a Ty tag (Fig. 7A). The correct integration of the vector in the *RON10* locus was verified by PCR (Fig. 7B). Western-blot analyses of the *Δron10* parasites showed the absence of RON10 and the concomitant detection of two lower bands with anti-Ty antibodies that were not detected in the control strain (Fig. 7C). Analysis of the *Δron10* parasites by IFA revealed that in the absence of RON10, most parasites did not show any detectable RON9 labeling. When detected, RON9 was not located in the rhoptry neck but instead showed a peri-nuclear labeling reminiscent of what we observed for RON10 in the absence of RON9, suggesting that similarly, RON9 did not traffic beyond the ER without its partner RON10 (Fig. 7D). Therefore, RON9 and RON10 form a necessary complex while trafficking to the rhoptries and the absence of one prevents the correct routing of its partner.Because *RON9* deletion resulted in defective targeting of RON10 and subsequent degradation of the RON10 partner, the *Δron9* knock-out parasite is likely a functional RON9 and RON10 knockout and was thereafter referred to as RON9-RON10KO.

### RON9-RON10KO parasites exhibit normal rhoptry morphology, normal invasion and intracellular replication *in vitro*, and normal virulence in a mouse model

In order to determine whether the deletion of RON9 and the absence of RON10 from rhoptries had any effect on rhoptry structure, we analyzed the parasites by electron microscopy. This study did not reveal any differences in the electron density or ultrastructure of the rhoptries, indicating that the RON9-RON10 complex is not a key structural determinant for defining these features (data not shown).

**Figure 8:**
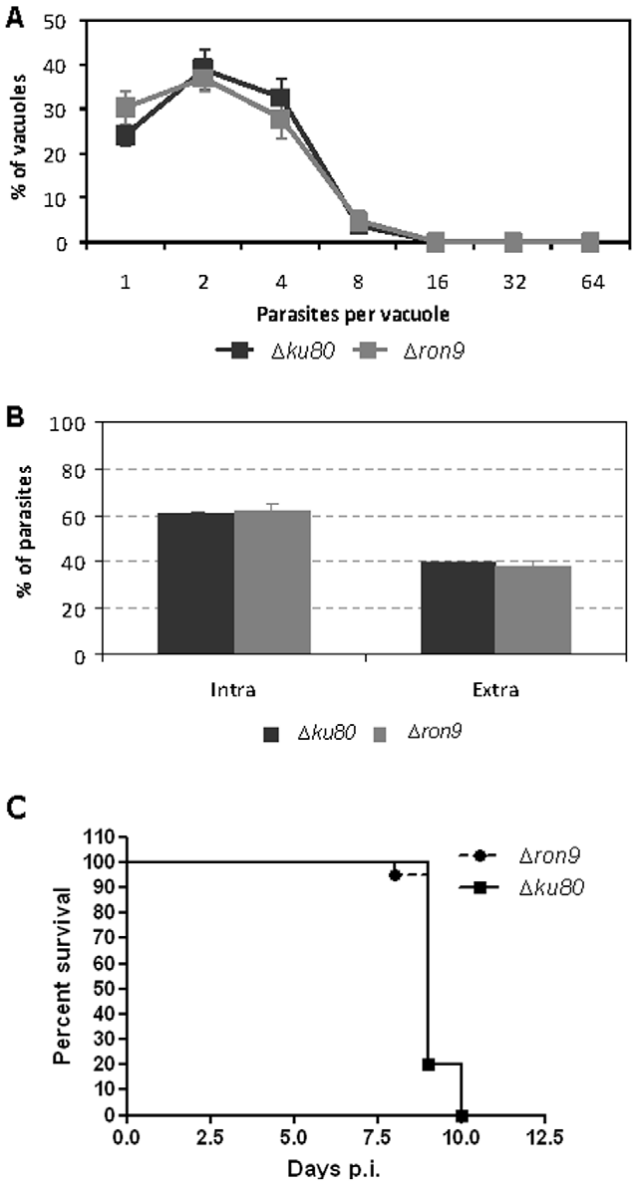
*Δron9* parasites do not display any defect in replication and invasion *in vitro*, nor in virulence *in vivo*. (A) The intracellular replication of *Δron9* parasites was compared to that of the parental *Δku80*, 18 h post-infection. For this, the number of parasites per vacuole was counted after anti-SAG1 labeling of the parasite surface. Values represent means ± standard deviations (SD), n = 3, of 4 independent assays. (B) Invasion assays were carried out on freshly released highly synchronized tachyzoites. Invasion was allowed to take place for 5 min prior cells fixation and IFA processing as described in material and methods section. Values represent means ± standard deviations (SD), n = 3, from a representative experiment out of 3 independent assays. (C) Deletion of *RON9* did not reduce virulence in mice. Twenty tachyzoites of the indicated strains were injected i.p. into Balbc mice (*n* = 20), and mouse survival was monitored daily for 15 d.

We then assessed the replication rate of the RON9-RON10KO parasites as compared to the parental *Δku80*. To this end, HFFs were infected for 18 h prior to formaldehyde fixation and counting of the number of parasites per vacuoles. Our results showed no significant differences between the parental strain and the RON9-RON10KO parasites (Fig. 8A). Next, we compared the invasion capacity of the RON9-RON10KO strain with that of the parental *Δku80* and observed no significant differences in the number of intracellular or extracellular parasites, thus indicating that the deletion of RON9 did not affect the invasion capacity of the parasites (Fig. 8B).

It has already been described that knock-out parasites, despite displaying a normal invasion capacity *in vitro*, might be impaired in their virulence *in vivo* [35]. Therefore, the two strains were compared for their virulence in mice. All the parasites used in this study were of the RH strain, a type 1 strain, which typically kills mice 7–10 d after i.p. infection with a single tachyzoite. As presented in Fig. 7C, all mice died between days 8 and 10 after infection with 20 parasites, indicating that the lack of RON9-RON10 complex does not decrease virulence in mice.

## Discussion

In the past few years, rhoptry proteins specifically located in the rhoptry neck have been described and shown to take part in the formation of the so-called moving junction, a close apposition of the plasma membranes of both the parasite and its host cell, which allows the parasite to actively propel itself into the nascent parasitophorous vacuole. Here, we report the characterization of two novel rhoptry neck proteins, RON9 and RON10 that form a new complex independent of the MJ complex. In addition, we were unable to detect this complex at the MJ. The rhoptries are secretory organelles that inject proteins into the host cell where RONs have been so far shown to associate to the host cell membrane part of the MJ (RON2/RON4/RON5/RON8) while ROPs are injected into the host nucleus (ROP16 [19], PP2C [36]), the host cytosol (Toxofilin [37], ROP13 [23]) or associated to the nascent PV membrane (ROP2 family including ROP18 and ROP5 [18], [38], [39]. Though clearly located in the rhoptry neck in intracellular parasites, RON9 and RON10 could not be detected after invasion, when rhoptries are discharged in the host cell. The absence of detection out of the parasite could be due either to an excessive dilution impairing detection using antibodies or to the possibility that RON9 and RON10 might be structural components of the rhoptry neck not secreted during invasion. This hypothesis still requires further investigations.

We have demonstrated that RON9 and RON10 form a highly stable complex. In the absence of RON9, RON10 is retained in the ER in parasites that undergo endodyogeny and is further degraded during the end of the cell cycle. Similar observations for RON9 were made when RON10 was disrupted. These data strongly argue for an interaction between RON9 and RON10 during their trafficking through the secretory pathway en route for the rhoptries. The fact that in the absence of one partner, the other one was found neither in the Golgi, nor in the PV space or at the plasma membrane but instead is stacked in the ER in dividing parasite and disappeared after division, might indicate that RON9 and RON10 do not reach the Golgi because they are not correctly folded (with which it forms a disulfide bridge hetero-complex), and then are eliminated by a retrieval transfer to a degradation compartment. Protein-protein interaction required for proper trafficking to rhoptry has already been described in *Plasmodium* [40] but is the first example in *T. gondii*. Truncation of the C-terminus of the rhoptry protein RAP1 (rhoptry associated protein 1) results in disruption of its interaction with RAP2/RAP3 (rhoptry associated proteins 2 and 3) with which it forms a low molecular weight complex and causes RAP2 (and probably RAP3) to be retained in the ER [40].

RON9 contains a set of repetitions enriched in proline (P), glutamic acid (D), aspartic acid (E) and serine (S) or threonine (T) typical of PEST sequences. These sequences are targets for rapid degradation and it is well known that caspase cleavage sites are commonly found within PEST motifs [41]. It is proposed that after cleavage of a PEST sequence, the exposed termini serve as unstructured initiation site for ubiquitin-proteasome dependent and independent degradation, and this would explain the inherent susceptibility to proteolysis among proteins containing PEST motifs. Considering RON9, we suggested that the PEST repetitions would be also subjected to proteolysis and that the successive digestions of the PEST sequences would explain the presence of lower bands of decreasing intensity and regular spacing present under the major band corresponding to RON9 in western blot. Whether RON9 is subjected *in vivo* to caspase degradation or whether these bands correspond to degradation after lysis remains to be determined. RON9 also contains a Sushi domain (or CCP for complement control protein). This domain is also present in TgRON1 [42], which is the ortholog of the *P. falciparum* apical sushi protein *Pf*ASP1 [42], also detected in the rhoptry neck [43]. Finally, RON9 contains ankyrin repeats, which are the most common protein–protein interaction motif in nature, and is predominantly found in eukaryotic proteins but also in pathogenic or symbiotic bacterial pathogens which deliver ANK-containing proteins into eukaryotic cells through secretion systems, where they mimic or manipulate various host functions. The ankyrin repeat is a 33-residues motif that often occurs in tandem arrays, which cooperatively fold into structures that mediate molecular recognition via protein–protein interactions [44] and are involved in many cellular functions in eukaryotes, such as inhibition or development of tumors, transcriptional regulation, cell cycle, oncogenesis, signal transduction, and modulation of the inflammatory response mediated by NF-kB [28]. Genomic analysis of viruses that infect eukaryotic cells has also revealed a large number of *ank* genes [45] where they are implicated in host cell tropism and permissiveness [46]. The presence of such repeats in RON9 suggests a potential interaction with host cell proteins upon invasion that remains to be explored.

In order to address the function of RON9/RON10 complex in *T. gondii*, we have generated *Δron9* parasites, which, based on RON10 mis-localization and degradation observations, likely corresponds to a RON9/RON10^−^ phenotype. These parasites were not altered in HFF invasion capacity *in vitro*, neither in replication and tachyzoite virulence *in vivo*. Since neither RON9 nor RON10 are conserved across the Apicomplexa phylum, we had assumed that these proteins might not be involved in a crucial conserved mechanism shared by apicomplexan parasites. However, homologues of RON9 and RON10 have been detected in *Eimeria spp.*, which could highlight a more specialized function for these proteins in the *Coccidia*. Orthologues have been also found in *Cryptosporium spp.*, another apicomplexan parasite of humans and other vertebrates. *Cryptosporidium* zoites attach to the apical membrane of host cells but, in contrast to other apicomplexans, do not form a moving junction and do not invade the cytoplasm of the host cells, but induce host-cell membrane protrusion that encapsulates the parasite [47]. Therefore *Cryptosporidium* resides within a PV that has an extracytosolic location between the apical plasma membrane and the host cytoplasm, where a zone of tight contact is observed. In addition, and in striking contrast with other Apicomplexa, *Cryptosporidium* induces the recruitment and remodeling of host cell actin at the site of entry [48]. According to these major differences, the conserved AMA1/RON2/RON4/RON5 complex is absent from the *Cryptosporidium* genome [49]. The conservation of RON9-RON10 in the genome of *Cryptosporium* therefore further argues against a participation of this complex in the MJ formation. The primary site of infection for *Coccidia*, as well as *Cryptosporidium* spp., is the epithelial cells of the gastrointestinal tract. The RON9-10 complex might therefore be linked to the interaction with a brush border membrane, which may need to be locally disorganized by the parasite before building the PV. Another possibility could be that secretion of the RON9-RON10 complex into the host cell involves molecular mimicry of host proteins to modulate specific epithelial cell processes as for pathogenic bacteria with ankyrin-containing proteins. Alternatively, RON9 and RON10 could be involved in cell and tissue tropism by driving a specific interaction between these Apicomplexa and the epithelial cells of the gastrointestinal tract.

## Materials and Methods

### Strains, culture

Tachyzoites of the RH *hxgprt*- strain of *T. gondii* deleted for hypoxanthine guanine phosphoribosyl transferase (*ΔHX* strain) [50] or RH *KU80*- deleted for the *KU80* gene *Δku80* strain) [51] were used throughout the study. Parasites were maintained on human foreskin fibroblasts (HFFs) in RPMI medium (Gibco BRL) supplemented with 5% foetal calf serum (FCS), 1% glutamine and 1% penicillin-streptomycin. The monocytic cell line THP-1 (ATCC TIB-202) was maintained as suspension culture in RPMI 1640 medium (Invitrogen), supplemented with 10% FCS.

For *T. gondii* transfections, 1,5.10^7^ extracellular tachyzoites were collected by centrifugation, washed once in cytomix buffer [52] and resuspended in 800 µl of cytomix supplemented with 3 mM ATP and 3 mM reduced glutathione. 20–80 µg of DNA was used for each transfection. The electroporation conditions used were as follows: 2.02 kV, 25 µF, 50 Ω. Following electroporation, transfected parasites were immediately deposited onto HFFs monolayers in fresh complete medium. To select for recombinant parasites, mycophenolic acid (20 µg/ml) and xanthine (50 µg/ml) or pyrimethamine at 1 µM were added in the medium.

### Molecular biology

Total RNA was extracted from *T. gondii* tachyzoites using RNA extraction kit (Qiagen). cDNA amplification was performed using the Superscript first strand synthesis kit (Invitrogen) as described by the manufacturer. cDNA fragments of TGME49_108600, TGME49_108710 and TGME49_061750 were amplified using the high fidelity Phusion polymerase (Finnzymes) and primers ML174 to ML177, ML200 to ML203, ML493, ML494, and ML552 to ML554 (Table S1), and further cloned into the pCR-Blunt II-TOPO vector (Invitrogen). After sequencing, the complete open reading frame of RON9 and RON10 was reconstituted from the overlapping cDNA sequences.

To engineer a RON10-HA strain, the C-terminal part of *RON10* was cloned in the pHA3-LIC-DHFR vector using the ligation independent cloning procedure and primers ML420 and ML421 (Table S1), as described previously [51]. The resulting vector was linearized with SnaBI prior to parasite transfection. Correct insertion at the endogenous locus was verified by PCR using primers ML176 and ML292.

For the *Δron9* construct, 5′ and 3′flanking regions of 1992 bp and 2011 bp respectively of *RON9* were amplified by PCR from *T. gondii Δhxgprt* gDNA using primers ML439 to ML442 (Table S1). 5′ and 3′ PCR fragments were cloned subsequently in the pminiHXGPRT plasmid [50] in NotI/BamHI and HindIII/KpnI sites respectively. The resulting vector was linearized with KpnI prior to parasite transfection.

To engineer a *Δron10* strain, an internal 1000 bp region of *RON10* was amplified by PCR using primers ML875 and ML876 (Table S1) and subsequently cloned KpnI/NsiI into pTUB8MycGFPPfMyoAtailTy-HX [53]. Following linearization with BstEII, the vector was transfected in *T. gondii* parasites.

### Immuno-affinity purification, and Western blotting

To identify the complex immuno-purified by mAb 2A7, mAb immunoglobulins were purified from ascitic fluid by affinity chromatography on protein B Sepharose 4B and cross-linked to CnBR-activated Sepharose 4B (GE Healthcare), then immuno-affinity purification was done as described previously [10]. For all the other immuno-affinity purifications, antibodies were bound to 20 µl of protein G-sepharose (Amersham). Binding was allowed to proceed for 2 h in 1 ml PBS, followed by 3 washes in 0.1 M Tris-HCl pH 8.0, 1 M NaCl. Immuno-precipitations were performed with 5.10^8^ parasites solubilized in lysis buffer (50 mM Tris-HCl pH 8.3, 150 mM NaCl, 4 mM EDTA, 1 mM PMSF, 1% Nonidet 40 (NP40)) for 1 h at 4°C. The lysate was centrifuged 1 h at 12000 *g* and the supernatant was incubated overnight with immunosorbents at 4°C under gentle agitation. Immunosorbents were then washed 5 times in washing buffer 1 (50 mM Tris-HCl pH 8.3, 1 M NaCl, 0.5% NP40), and once in washing buffer 2 (5 mM Tris-HCl pH 6.8). Bound antigens were eluted during 5 min in SDS-PAGE loading buffer at 95°C and subjected to electrophoresis.

Proteins were separated by SDS-PAGE in the presence (reduced) or absence (non-reduced) of 100 mM DTT, transferred to nitrocellulose and subjected to Western blot analysis as previously described [26].

### Mass spectrometry and bioinformatic analyses

Immunoprecipitated proteins were resolved by SDS-PAGE, stained with colloidal Coomassie blue, excised and digested with trypsin (sequencing grade, Promega, Madison, WI), as described [54]. Samples were analysed by nanoflow HPLC-nano-electrospray ionization on a Bruker Esquire 3000+ion trap (Bremen, Germany) coupled with an LC-Packings HPLC (Amsterdam, the Netherlands) as described previously [10]. All MS/MS spectra were searched against the *T. gondii* entries of Swiss-Prot and Trembl databases (http://www.expasy.ch), or ESTs and genomic release of ToxoDB 3.0 database (http://www.toxodb.org) (Kissinger *et al.*, 2003) by using the Mascot v 2.0 algorithm (http://www.matrixscience.com). All significant hits were manually inspected.

Sequences analyses were performed using SMART (http://smart.embl-heidelberg.de/), Pfam (http://pfam.sanger.ac.uk/search?tab=searchSequenceBlock/), Prosite (http://www.expasy.ch/tools/scanprosite/), SignalP (http://www.cbs.dtu.dk/services/SignalP/), TMpred (http://www.ch.embnet.org/software/TMPRED_form.html), Radar (http://www.ebi.ac.uk/Tools/Radar/index.html), PestFind (http://emboss.bioinformatics.nl/cgi-bin/emboss/epestfind), and CLUSTAL (http://www.ebi.ac.uk/Tools/msa/clustalw2/) bioinformatic programs.

### Immunofluorescence assay (IFA) and electron microscopy

Cells were fixed in methanol for 7 min or in 4% paraformaldehyde (PAF) for 30 min, washed and permeabilized with 0.1% Triton ×100 or with 0.05% saponin in PBS for 10 min. Saturation was performed in PBS supplemented with 10% FCS (PBS-10%FCS) for 1 h. Primary antibodies were diluted in PBS-2% FCS before addition to the cells and further incubated for 1 h. After 3 washes in PBS, cells were incubated with Alexa conjugated secondary antibodies (Sigma) diluted in PBS-2% FCS. When required, nuclei were stained with Hoechst. Finally the coverslips were washed and mounted onto microscope slides using Immumount (Calbiochem). Images were collected either i) with a Leica DMRA2 microscope equipped for epifluorescence, the images being recorded with a COOLSNAP CCD camera (Photometrics) driven by the Metaview software (Universal Imaging Co.) or ii) with a Zeiss Axioimager epifluorescence microscope and images were recorded with a Zeiss Axiocam MRm CCD camera driven by the Axiovision software (Zeiss), at the Montpellier RIO imaging facility. The antibodies dilutions used in this study were as follows: mouse mAb 2A7 (1∶200) [24], rabbit anti-RON2-4 (1∶500) [13] mouse or rabbit anti-HA (ClonTech, 1∶200), rat anti-RON9 (1∶200) (this study), rabbit anti-PEST (1∶200) (this study), rat anti-RON10 (1∶200) (this study), rabbit anti-IMC1 (1∶1000) [55], mouse anti-ISP1 (1∶1000) [32], mouse mAb T4 2E5 anti-SAG1 (1∶1000) [56], rabbit anti-ROP1 (1∶1000) (J.F. Dubremetz and O. Mercereau-Puijalon, unpublished results).

For immunoelectron microscopy, tachyzoites infected cells were fixed with 4% formaldehyde and 0.05% glutaraldehyde in PBS for 15 min, dehydrated in ethanol and embedded in LR White (London Resin Co.). Thin sections were collected on parlodion-carbon-coated nickel grids and floated for 30 min on 2.5% non fat dry milk and 0.1% Tween-20 in PBS (PBS-milk-Tween). The grids were saturated for 1 h with 500 µg protein A/ml PBS, transferred successively for 2 h each on dilutions of mouse ascitic fluid, followed by anti-mouse IgG-IgM rabbit antibodies, and by 10 nm protein A-gold in PBS-milk-Tween. Sections were stained with 4% uranyl acetate in water and observed with a Hitachi H600 microscope.

Cryoimmuno electronmicroscopy: HFF infected for 18 h with tachyzoites were fixed with 4% paraformaldehyde-0.05% glutaraldehyde in 0.2 M phosphate buffer pH 7.4 for 90 min. The monolayer was then scraped off the bottom of the dish and pelleted. The pellet was infiltrated with a solution containing 2.3 M sucrose-10% polyvinyl pyrrolidone in 0.1 M phosphate buffer pH 7.4 for 4 h and then frozen in liquid nitrogen. Sections were made at −100°C with a Leica Ultracut equipped with an FC4 attachment and were collected on 10% FCS in PBS (PBSS). Immunolocalization was performed by successive incubations of the sections with ascitic fluid, rabbit anti-mouse IgG, and 10 nm Protein A-gold in PBSS. The grids were then stained with a methyl cellulose uranyle acetate mixture and observed with a Jeol 1200 EX microscope.

### Recombinant proteins and specific anti-sera production

DNA sequences corresponding to amino acids 908 to 1203 or 350 to 687 of RON9 (Genbank JQ655737) and RON10 (Genbank JQ655738) proteins respectively were obtained by PCR from *T. gondii* tachyzoites cDNA with primers ML174/ML175 and ML176/ML177 respectively. They were then subcloned into pCR-Blunt II-TOPO vector and further cloned into the BamHI and Xho1 sites of pGEX-4T-3 (GE healthcare). Following transformation into *E. coli* C41 cells, the synthesis of recombinant GST-tagged RON9 and RON10 proteins was induced with 0.5 mM isopropyl 1-thio-D-galactopyranoside for 2 h at 37°C. The bacterial pellet was dispersed in PBS containing 2 mM EDTA and 2 mM PMSF, and supplemented with a complete mixture of protease inhibitors (Roche Applied Science). Cells were broken using a French press (Thermo Spectronic) at 10,000 p.s.i., then centrifuged at 7.700 g for 20 min at 4°C. The fusion proteins were purified by electro-elution after SDS–PAGE under denaturing conditions.

Rats were immunized twice two weeks apart with 100 µg proteins in Freund's complete adjuvant and boosted with two successive injections of 50 µg proteins in incomplete adjuvant at two weeks intervals. Pre-immune sera were collected prior to the first immunization and rat anti-RON9 and anti-RON9HP (anti-RON10) sera were collected 3 weeks after the final boost and subsequently analyzed by IFA and immunoblot.

A 24 residues peptide (QANASQSSETPAEEPKQAEE) comprising the entire repeat sequence of RON9 was synthesized by Covalab (Villeurbane, France), and used to immunize rabbits and generate anti-PEST serum.

### *In vitro* and *in vivo* phenotypic characterization of RON9-RON10KO parasites

To assess *T. gondii* intracellular replication, 2.10^5^ tachyzoites were inoculated onto HFFs monolayers in a 24 wells plate and parasites replication was stopped 18 h or 24 h post-infection by fixation in 4% PAF. To facilitate parasites counting, infected cells were permeabilized with saponin and labelled with anti-SAG1 antibody. The number of parasites per vacuole was counted under the microscope to measure intracellular replication.

For invasion assays, 5.10^6^ freshly released tachyzoites were synchronized using a K^+^ buffer shift [57] during 20 min at 37°C and subsequently allowed to invade for 5 min in invasion buffer. Invasion was stopped by fixation in 4% PAF and parasites were further processed for IFA. Prior to triton permeabilization, extracellular parasites were labelled with anti-SAG1 antibodies, while following permeabilization, intracellular parasites were stained with anti-ROP1 antibodies. All the subsequent steps were performed as described above in the IFA section.

To assess the virulence *in vivo*, 20 tachyzoites were injected i.p. into 20 female BALB/c mice at 8 weeks of age. Survival of the mice was checked daily. In order to monitor an equal viability of tachyzoites from the KO and WT strains, samples of the parasites used for infection were used to infect THP-1 cells at a ratio of 5 cells per parasite and incubated at 37°C for 24 h. Parasite multiplication was assessed by DNA extraction and real-time PCR of a *T. gondii* specific sequence.

### Ethics statement

This study was conducted according to European Union guidelines for the handling of laboratory animals and the immunization protocol for antibody production in rats was conducted at the animal house of the Centre de Recherche de Biochimie Macromoléculaire (Montpellier) and approved by the Committee on the Ethics of Animal Experiments (Languedoc-Roussillon, Montpellier) (Permit Number: D34-172-4, delivered on 20/09/2009).

### Statistical analysis

Statistical analyses were performed in GraphPad Prism 4 for Windows, with Students's *t*-test (unpaired, equal variance, two-tailed test) for comparisons with data that fit a normal distribution. Survival of mice was represented as Kaplan-Meier plot using GraphPad Prism 5. Levels of significance were determined with the Logrank test using GraphPad.

## Supporting Information

Figure S1 **TgRON9 protein sequence following cDNA sequencing.** Specific sequences or domains identified by bioinformatic analyses include the N-terminal signal peptide (pink), 12 PEST repetitions (light blue), 6 ankyrin domains (light red), 1 Sushi domain (blue) and 1 putative transmembrane domain at the extreme C-terminus (red). The TgRON9 protein sequence used to generate anti-RON9rec antibodies corresponds to the grey bar, while the peptides leading to TgRON9 identification by mass-spectrometry are shown in green.
(PDF)

Figure S2 **TgRON10 protein sequence following cDNA sequencing.** Bioinformatic analyses led to the identification of a signal peptide in the N-terminus of RON10 (pink), The TgRON10 protein sequence used to generate anti-RON9HP (or anti-RON10) antibodies is highlighted in grey, while the single peptide leading to TgRON10 identification by mass-spectrometry is shown in green.
(PDF)

Figure S3 **IFA localization of RON9HP on intracellular** ***Δhxgprt*** **parasites fixed with 4% PAF using anti-RON9HP serum.** Partial co-localization of mAb 2A7 and anti-RON9HP labeling was observed, suggesting a possible rhoptry neck localization of RON9HP.
(PDF)

Figure S4 **Protein alignment of RON9 orthologues.** BLAST analysis of TgRON9 protein sequence revealed RON9 orthologues in *Eimeria tenella* (EtRON9), *Neospora caninum* (NcRON9) and *Cryptosporidium parvum* (CpRON9). Amino-acid conservation between the different species is highlighted in grey and black. TgRON9 signal peptide, ankyrin and sushi domains and transmembrane domain are shown by blue arrows on top of the alignment. The ankyrin domain that is not conserved in CpRON9 is shown in light red.
(PDF)

Figure S5 **Search for repetitions in RON9 and RON10 orthologues using Radar program (** http://www.ebi.ac.uk/Tools/Radar/index.html **) led to the identification of 12 repeats of 21 bp in TgRON9, 14 repeats of 34 bp in NcRON9 and 5 repeats of 29 bp in CpRON10.**
(PDF)

Figure S6 **Protein alignment of RON10 orthologues, including sequences of** ***T. gondii*** **(TgRON10),** ***N. caninum*** **(NcRON10) and** ***C. parvum*** **(CpRON10).** Amino-acid conservation between the different species is highlighted in grey and black.
(PDF)

Figure S7 **Generation of** ***Δron9*** **-R10HA parasites.** (A) PCR reactions to check for replacement of *RON9* gene with *HXGPRT* as shown in figure 5A were performed on gDNA of RON10HA (lane 1) or *Δron9*-R10HA parasites (lane 2). Correct integration of the vector was verified on the 5′ (p5′) and 3′ (p3′) side of the recombination event. PCR amplificatin of the *ATG3* gene was used as a control of gDNA integrity. As expected, DNA fragments were amplified from the *Δron9*-R10HA population with the integration PCRs while no DNA could be amplified from the parental strain. Primers in *T. gondii ATG3* gene allowed DNA amplification for the 3 gDNAs tested. (B) Western-blot performed on RON10HA (lane 1) or *Δron9*-R10HA (lane 2) lysates shows that RON9 is not detected in the *Δron9*-R10HA parasites using mAb 2A7 (anti-RON9), while RON10 and RON4 are revealed with anti-HA and mAb 4H1 respectively in the *Δron9*-R10HA parasites.
(PDF)

Table S1 **Primers used in this study.**
(XLSX)
