# Supplementary figures and images for: Identification of a New Rhoptry Neck Complex RON9/RON10 in the Apicomplexa Parasite Toxoplasma gondii
Source: PLoS One. 2012 Mar 12;7(3):e32457. doi: 10.1371/journal.pone.0032457 (PMC3299665; doi:10.1371/journal.pone.0032457)

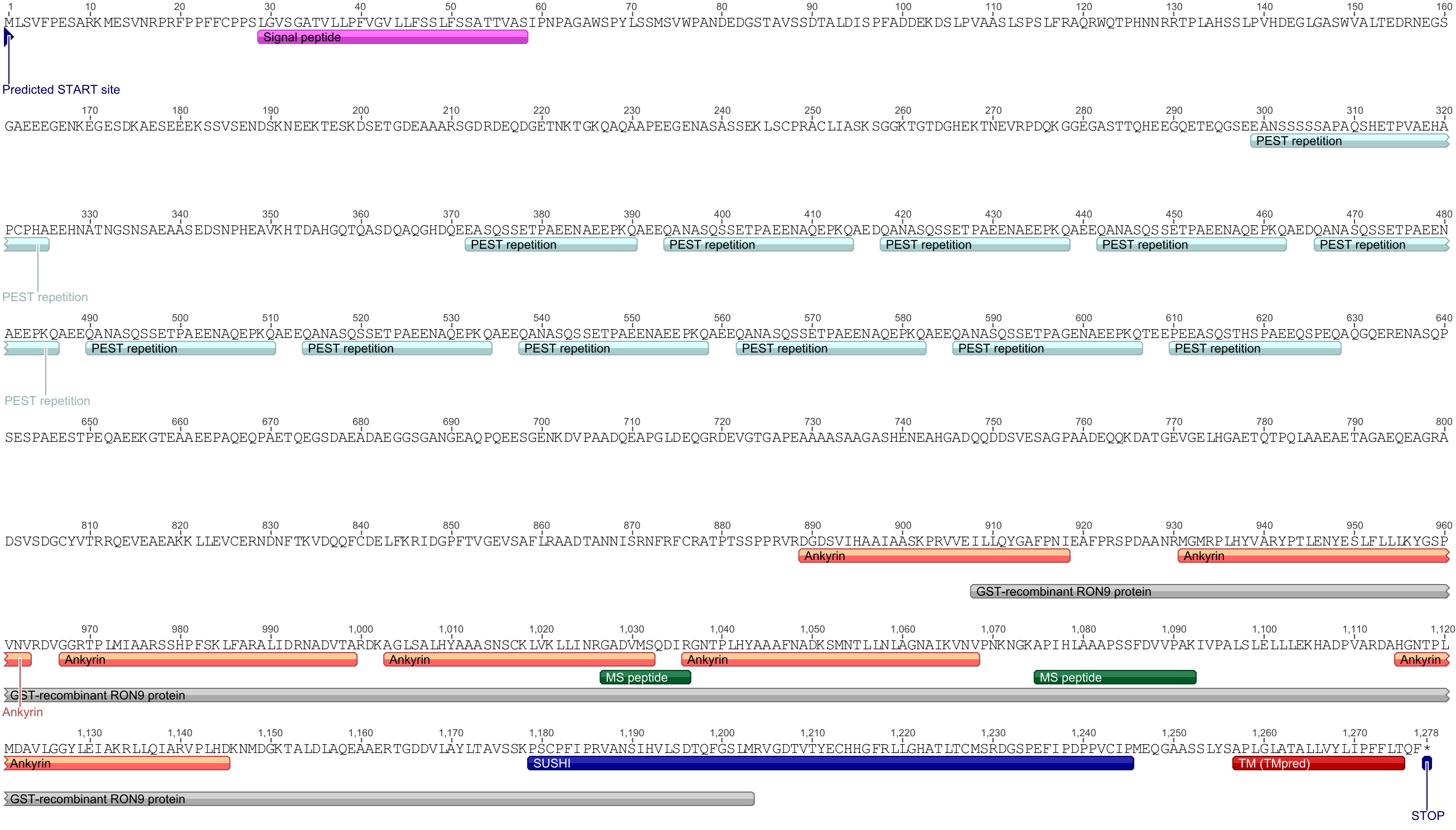

Supplement: Figure S1 — TgRON9 protein sequence following cDNA sequencing. Specific sequences or domains identified by bioinformatic analyses include the N-terminal signal peptide (pink), 12 PEST repetitions (light blue), 6 ankyrin domains (light red), 1 Sushi domain (blue) and 1 putative transmembrane domain at the extreme C-terminus (red). The TgRON9 protein sequence used to generate anti-RON9rec antibodies corresponds to the grey bar, while the peptides leading to TgRON9 identification by mass-spectrometry are shown in green. (PDF) [file pone.0032457.s001.pdf]

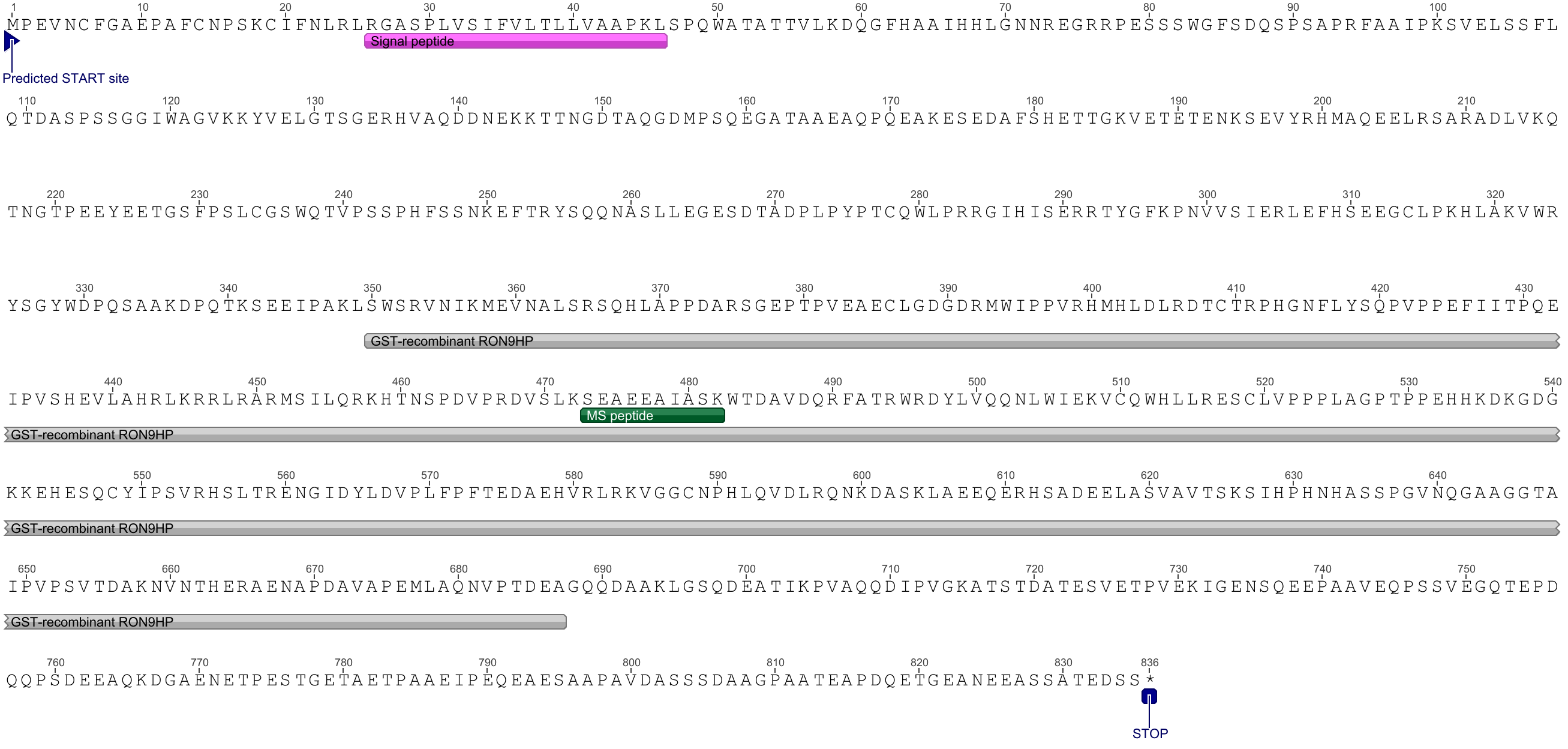

Supplement: Figure S2 — TgRON10 protein sequence following cDNA sequencing. Bioinformatic analyses led to the identification of a signal peptide in the N-terminus of RON10 (pink), The TgRON10 protein sequence used to generate anti-RON9HP (or anti-RON10) antibodies is highlighted in grey, while the single peptide leading to TgRON10 identification by mass-spectrometry is shown in green. (PDF) [file pone.0032457.s002.pdf]

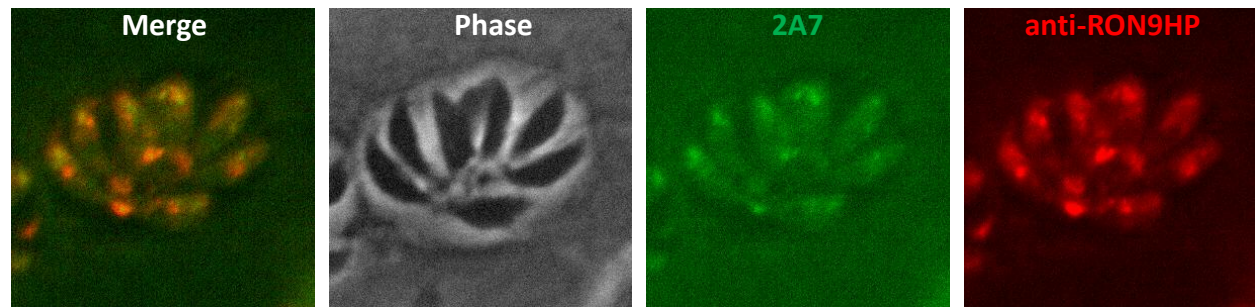

**Figure S3**

Supplement: Figure S3 — IFA localization of RON9HP on intracellular Δhxgprt parasites fixed with 4% PAF using anti-RON9HP serum. Partial co-localization of mAb 2A7 and anti-RON9HP labeling was observed, suggesting a possible rhoptry neck localization of RON9HP. (PDF) [file pone.0032457.s003.pdf]

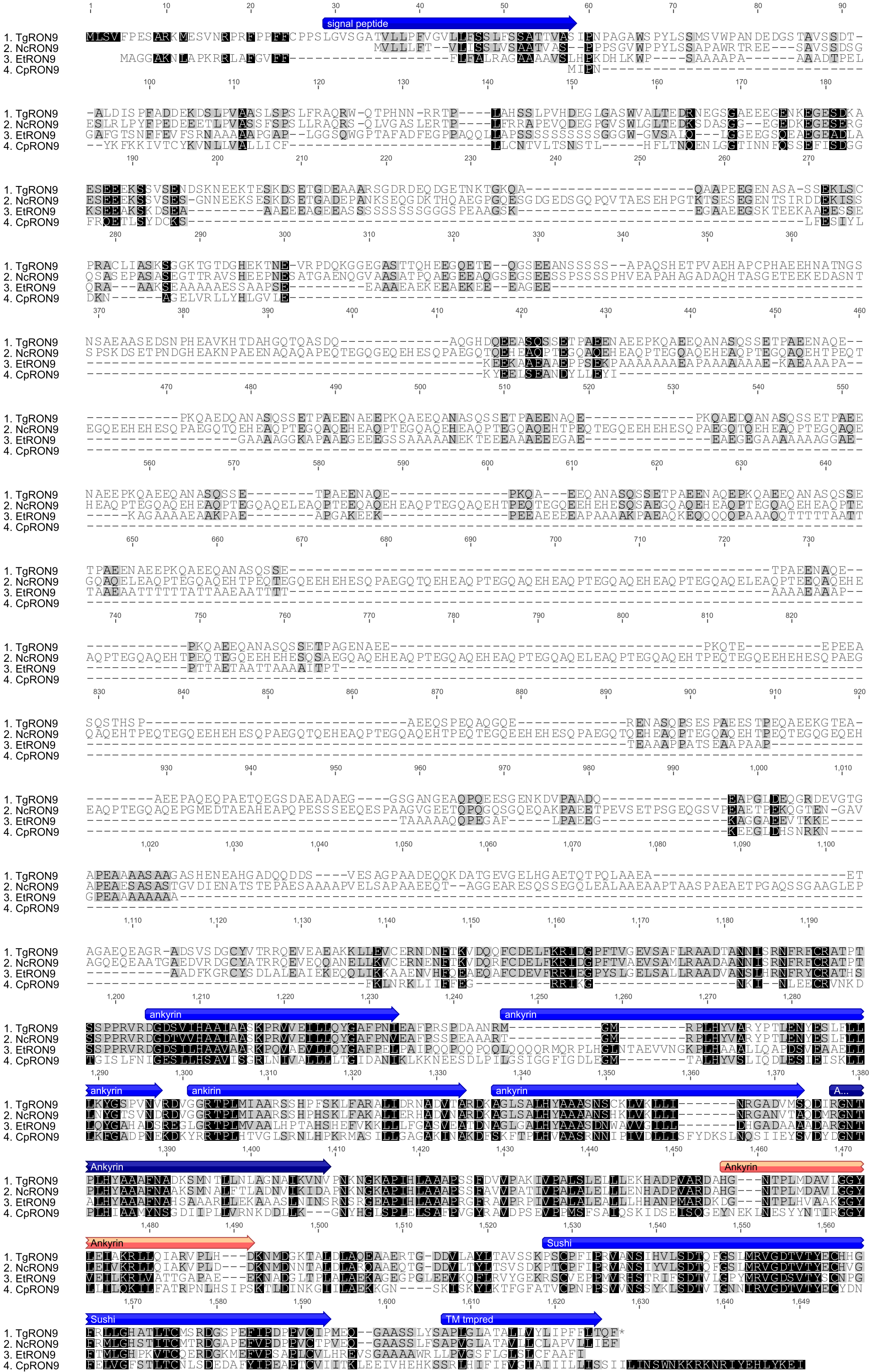

Supplement: Figure S4 — Protein alignment of RON9 orthologues. BLAST analysis of TgRON9 protein sequence revealed RON9 orthologues in Eimeria tenella (EtRON9), Neospora caninum (NcRON9) and Cryptosporidium parvum (CpRON9). Amino-acid conservation between the different species is highlighted in grey and black. TgRON9 signal peptide, ankyrin and sushi domains and transmembrane domain are shown by blue arrows on top of the alignment. The ankyrin domain that is not conserved in CpRON9 is shown in light red. (PDF) [file pone.0032457.s004.pdf]

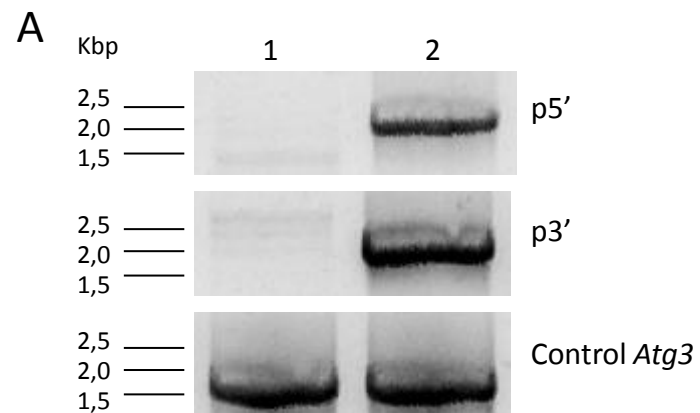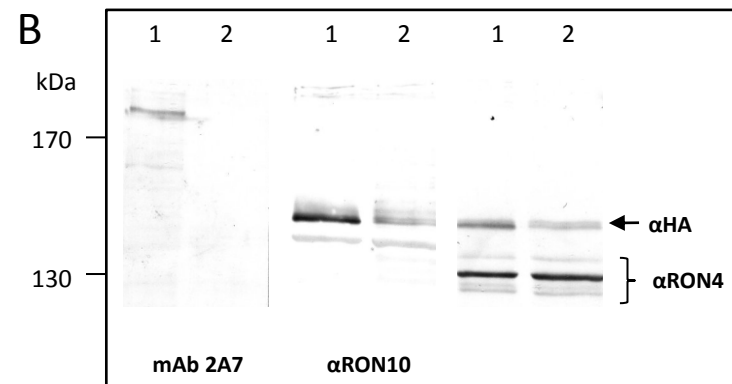

**Figure S7**

Supplement: Figure S7 — Generation of Δron9 -R10HA parasites. (A) PCR reactions to check for replacement of RON9 gene with HXGPRT as shown in figure 5A were performed on gDNA of RON10HA (lane 1) or Δron9-R10HA parasites (lane 2). Correct integration of the vector was verified on the 5′ (p5′) and 3′ (p3′) side of the recombination event. PCR amplificatin of the ATG3 gene was used as a control of gDNA integrity. As expected, DNA fragments were amplified from the Δron9-R10HA population with the integration PCRs while no DNA could be amplified from the parental strain. Primers in T. gondii ATG3 gene allowed DNA amplification for the 3 gDNAs tested. (B) Western-blot performed on RON10HA (lane 1) or Δron9-R10HA (lane 2) lysates shows that RON9 is not detected in the Δron9-R10HA parasites using mAb 2A7 (anti-RON9), while RON10 and RON4 are revealed with anti-HA and mAb 4H1 respectively in the Δron9-R10HA parasites. (PDF) [file pone.0032457.s007.pdf]
