# Supplementary material for: Identification of a New Rhoptry Neck Complex RON9/RON10 in the Apicomplexa Parasite Toxoplasma gondii
Source: PLoS One. 2012 Mar 12;7(3):e32457. doi: 10.1371/journal.pone.0032457 (PMC3299665; doi:10.1371/journal.pone.0032457)
Supplement: Figure S5 — Search for repetitions in RON9 and RON10 orthologues using Radar program ( http://www.ebi.ac.uk/Tools/Radar/index.html ) led to the identification of 12 repeats of 21 bp in TgRON9, 14 repeats of 34 bp in NcRON9 and 5 repeats of 29 bp in CpRON10. (PDF) [file pone.0032457.s005.pdf]

***T. gondii* RON9 repeats**

```
No. of Repeats: 12
Length: 21

Position in sequence
 -----------------------------------------------------------------------
  299-  325)    EANSSsssapaQSHETPVAEHAPCPHA
  372-  390     ..EAS......QSSETPAEENAEEPKQ
  394-  414)    QANAS......QSSETPAEENAQEPKQ
  418-  438     QANAS......QSSETPAEENAEEPKQ
  442-  462     QANAS......QSSETPAEENAQEPKQ
  466-  486     QANAS......QSSETPAEENAEEPKQ
  490-  510     QANAS......QSSETPAEENAQEPKQ
  514-  534     QANAS......QSSETPAEENAQEPKQ
  538-  558     QANAS......QSSETPAEENAEEPKQ
  562-  582     QANAS......QSSETPAEENAQEPKQ
  586-  606     QANAS......QSSETPAGENAEEPKQ
  610-  628     PEEAS......QSTHSPAEE..QSPEQ
------------------------------------------------------------------------
```

***N. caninum* RON9 repeats**

```
No. of Repeats: 14
Length: 34

Position in sequence
------------------------------------------------------------------------
  353-  389     QGeQEHESQPAEGQ.TQ.EHeaQP..TEGQ.AQ.EHEAQPTEG
  390-  426     QA.QEHEAQPTEGQ.AQ.EHtpEQ..TEGQ.EEhEHESQPAEG
  427-  462     QT.QEHEAQPTEGQ.AQ.EHeaQP..TEGQ.AQ.EHEAQPTEG
  463-  499     QA.QEHTPEQTEGQ.EEhEHesQP..AEGQ.TQ.EHEAQPTEG
  536-  572     QA.QEHEAQPTEGQ.AQ.EH..TPeqTEGQ.EEhEHESQSAEG
  573-  608     QA.QEHEAQPTEGQ.AQ.EHeaQP..TEGQ.AQ.ELEAQPTEG
  609-  645     QA.QEHTPEQTEGQeEH.EHesQP..AEGQ.TQ.EHEAQPTEG
  646-  681     QA.QEHEAQPTEGQ.AQ.EHeaQP..TEGQ.AQ.ELEAQPTEE
  682-  718     QA.QEHEAQPTEGQ.AQ.EH..TPeqTEGQ.EEhEHESQSAEG
  719-  742     QA.QEHEAQPTEGQ.AQ.EH..E........AQ......PTEG
  743-  779     QA.QELEAQPTEGQ.AQ.EH..TPeqTEGQ.EEhEHESQPAEG
  780-  804     QA.QEHTPEQTEGQ.E...............EH.EHESQPAEG
  805-  841     QT.QEHEAQPTEGQ.AQ.EHtpEQ..TEGQ.EEhEHESQPAEG
  842-  878     QT.QEHEAQPTEGQ.AQ.EHtpEQ..TEGQgEQ.EHEAQPTEG
------------------------------------------------------------------------
```

***Cryptosporidium parvum* RON10 repeats**

```
No. of Repeats: 5
Length: 29

Position in sequence
------------------------------------------------------------------------
   80-  108     KPEESNLDKTKPKEPSPDEKKPEESKSDE
  110-  138     KPGESKSDETKPGESKSDETKPGESKSDE
  140-  168     KPGESKSDETKPGESKSDETKPGESKSDE
  170-  198     KPEETKPDETKPEEAKSEEAKPEETKPDE
  200-  228     NPEESKPEETKPEEAKHDEENSGKGNSTE
```
