# Supplementary material for: Identification of a New Rhoptry Neck Complex RON9/RON10 in the Apicomplexa Parasite Toxoplasma gondii
Source: PLoS One. 2012 Mar 12;7(3):e32457. doi: 10.1371/journal.pone.0032457 (PMC3299665; doi:10.1371/journal.pone.0032457)
Supplement: Figure S6 — Protein alignment of RON10 orthologues, including sequences of T. gondii (TgRON10), N. caninum (NcRON10) and C. parvum (CpRON10). Amino-acid conservation between the different species is highlighted in grey and black. (PDF) [file pone.0032457.s006.pdf]

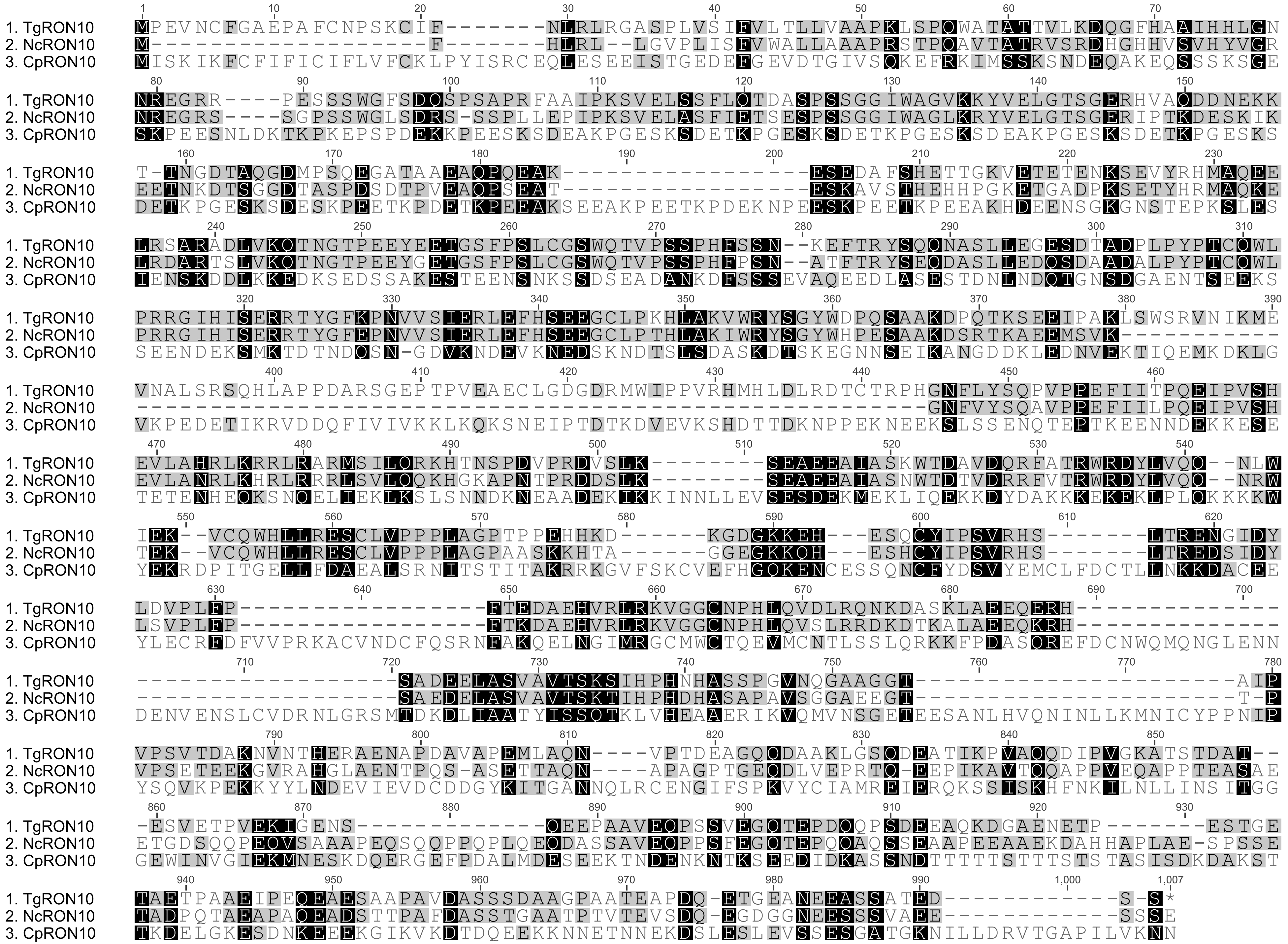

1 10 20 30 40 50 60 70
1. TgRON10 MPEVNCFGAEPAFCNPSKCIF-------NLRLRGASPLVSIFVLTLLVAAPKLSPQWATATTVLKDQGFHAAIHHLGN
2. NcRON10 M-------------------F-------HLRL--LGVPLISFVWALLAAAPRSTPQAVTATRVSRDHGHHVSVHYVGR
3. CpRON10 MISKIKFCFIFICIFLVFCKLPYISRCEQLESEEISTGEDEFGEVDTGIVSQKEFRKIMSSKSNDEQAKEQSSSKSGE
80 90 100 110 120 130 140 150
1. TgRON10 NREGRR----PESSSWGFSDQSPSAPRFAAIPKSVELSSFLQTDASPSSGGIWAGVKKYVELGTSGERHVAQDDNEKK
2. NcRON10 NREGRS----SGPSSWGLSDRS-SSPLLEPIPKSVELASFIETSESPSSGGIWAGLKRYVELGTSGERIPTKDESKIK
3. CpRON10 SKPEESNLDKTKPKEPSPDEKKPEESKSDEAKPGESKSDETKPGESKSDETKPGESKSDEAKPGESKSDETKPGESKS
160 170 180 190 200 210 220 230
1. TgRON10 T-TNGDTAQGDMPSQEGATAAEAQPQEAK-----------------ESEDAFSHETTGKVETETENKSEVYRHMAQEE
2. NcRON10 EETNKDTSGGDTASPDSDTPVEAQPSEAT-----------------ESKAVSTHEHHPGKETGADPKSETYHRMAQKE
3. CpRON10 DETKPGESKSDESKPEETKPDETKPEEAKSEEAKPEETKPDEKNPEESKPEETKPEEAKHDEENSGKGNSTEPKSLES
240 250 260 270 280 290 300 310
1. TgRON10 LRSARADLVKQTNGTPEEYEETGSFPSLCGSWQTVPSSPHFSSN--KEFTRYSQQNASLLEGESDTADPLPYPTCQWL
2. NcRON10 LRDARTSLVKQTNGTPEEYGETGSFPSLCGSWQTVPSSPHFPSN--ATFTRYSEQDASLLEDQSDAADALPYPTCQWL
3. CpRON10 IENSKDDLKKEDKSEDSSAKESTEENSNKSSDSEADANKDFSSSEVAQEEDLASESTDNLNDQTGNSDGAENTSEEKS
320 330 340 350 360 370 380 390
1. TgRON10 PRRGIHISERRTYGFKPNVVSIERLEFHSEEGCLPKHLAKVWRYSGYWDPQSAAKDPQTKSEEIPAKLSWSRVNIKME
2. NcRON10 PRRGIHISERRTYGFEPNVVSIERLEFHSEEGCLPTHLAKIWRYSGYWHPESAAKDSRTKAEEMSVK-----------
3. CpRON10 SEENDEKSMKTDTNDQSN-GDVKNDEVKNEDSKNDTSLSDASKDTSKEGNNSEIKANGDDKLEDNVEKTIQEMKDKLG
400 410 420 430 440 450 460
1. TgRON10 VNALSRSQHLAPPDARSGEPTPVEAECLGDGDRMWIPPVRHMHLDLRDTCTRPHGNFLYSQPVPPEFIITPQEIPVSH
2. NcRON10 --GNFVYSQAVPPEFIILPQEIPVSH
3. CpRON10 VKPEDETIKRVDDQFIVIVKKLKQKSNEIPTDTKDVEVKSHDTTDKNPPEKNEEKSLSSENQTEPTKEENNDEKKESE
470 480 490 500 510 520 530 540
1. TgRON10 EVLAHRLKRRLRARMSILQRKHTNSPDVPRDVSLK--------SEAEEAIASKWTDAVDQRFATRWRDYLVQQ--NLW
2. NcRON10 EVLANRLKHRLRRRLSVLQQKHGKAPNTPRDDSLK--------SEAEEAIASNWTDTVDRRFVTRWRDYLVQQ--NRW
3. CpRON10 TETENHEQKSNQELIEKLKSLSNNDKNEAADEKIKKINNLLEVSESDEKMEKLIQEKKDYDAKKKEKEKLPLQKKKKW
550 560 570 580 590 600 610 620
1. TgRON10 IEK--VCQWHLLRESCLVPPPLAGPTPPEHHKD------KGDGKKEH---ESQCYIPSVRHS-------LTRENGIDY
2. NcRON10 TEK--VCQWHLLRESCLVPPPLAGPAASKKHTA------GGEGKKQH---ESHCYIPSVRHS-------LTREDSIDY
3. CpRON10 YEKRDPITGELLFDAEALSRNITSTITAKRRKGVFSKCVEFHGQKENCESSQNCFYDSVYEMCLFDCTLLNKKDACEE
630 640 650 660 670 680 690 700
1. TgRON10 LDVPLFP-----------------FTEDAEHVRLRKVGGCNPHLQVDLRQNKDASKLAEEQERH--------------
2. NcRON10 LSVPLFP-----------------FTKDAEHVRLRKVGGCNPHLQVSLRRDKDTKALAEEQKRH--------------
3. CpRON10 YLECRFDFVVPRKACVNDCFQSRNFAKQELNGIMRGCMWCTQEVMCNTLSSLQRKKFPDASQREFDCNWQMQNGLENN
710 720 730 740 750 760 770 780
1. TgRON10 ------------------SADEELASVAVTSKSIHPHNHASSPGVNQGAAGGT----------------------AIP
2. NcRON10 ------------------SAEDELASVAVTSKTIHPHDHASAPAVSGGAEEGT----------------------T-P
3. CpRON10 DENVENSLCVDRNLGRSMTDKDLIAATYISSQTKLVHEAAERIKVQMVNSGETEESANLHVQNINLLKMNICYPPNIP
790 800 810 820 830 840 850
1. TgRON10 VPSVTDAKNVNTHERAENAPDAVAPEMLAQN----VPTDEAGQQDAAKLGSQDEATIKPVAQQDIPVGKATSTDAT--
2. NcRON10 VPSETEEKGVRAHGLAENTPQS-ASETTAQN----APAGPTGEQDLVEPRTQ-EEPIKAVTQQAPPVEQAPPTEASAE
3. CpRON10 YSQVKPEKKYYLNDEVIEVDCDDGYKITGANNQLRCENGIFSPKVYCIAMREIERQKSSISKHFNKILNLLINSITGG
860 870 880 890 900 910 920 930
1. TgRON10 -ESVETPVEKIGENS-------------QEEPAAVEQPSSVEGQTEPDQQPSDEEAQKDGAENETP-------ESTGE
2. NcRON10 ETGDSQQPEQVSAAAPEQSQQPPQPLQEQDASSAVEQPPSFEGQTEPQQAQSSEAAPEEAAEKDAHHAPLAE-SPSSE
3. CpRON10 GEWINVGIEKMNESKDQERGEFPDALMDESEEKTNDENKNTKSEEDIDKASSNDTTTTTSTTTSTSTASISDKDAKST
940 950 960 970 980 990 1,000 1,007
1. TgRON10 TAETPAAEIPEQEAESAAPAVDASSSDAAGPAATEAPDQ-ETGEANEEASSATED-------------S-S*
2. NcRON10 TADPQTAEAPAQEADSTTPAFDASSTGAATPTVTEVSDQ-EGDGGNEESSSVAEE-------------SSSE
3. CpRON10 TKDELGKESDNKEEEKGIKVKDTDQEEKKNNETNNEKDSLESLEVSSESGATGKNILLDRVTGAPILVKNN
